# Brain Delivery of Nanomedicines: Trojan Horse Liposomes for Plasmid DNA Gene Therapy of the Brain

**DOI:** 10.3389/fmedt.2020.602236

**Published:** 2020-11-16

**Authors:** William M. Pardridge

**Affiliations:** Department of Medicine, University of California, Los Angeles, Los Angeles, CA, United States

**Keywords:** blood-brain barrier, non-viral gene therapy, liposomes, nanoparticles, mnoclonal antibody, transferrin receptor, insulin receptor

## Abstract

Non-viral gene therapy of the brain is enabled by the development of plasmid DNA brain delivery technology, which requires the engineering and manufacturing of nanomedicines that cross the blood-brain barrier (BBB). The development of such nanomedicines is a multi-faceted problem that requires progress at multiple levels. First, the type of nanocontainer, e.g., nanoparticle or liposome, which encapsulates the plasmid DNA, must be developed. Second, the type of molecular Trojan horse, e.g., peptide or receptor-specific monoclonal antibody (MAb), must be selected for incorporation on the surface of the nanomedicine, as this Trojan horse engages specific receptors expressed on the BBB, and the brain cell membrane, to trigger transport of the nanomedicine from blood into brain cells beyond the BBB. Third, the plasmid DNA must be engineered without bacterial elements, such as antibiotic resistance genes, to enable administration to humans; the plasmid DNA must also be engineered with tissue-specific gene promoters upstream of the therapeutic gene, to insure gene expression in the target organ with minimal off-target expression. Fourth, upstream manufacturing of the nanomedicine must be developed and scalable so as to meet market demand for the target disease, e.g., annual long-term treatment of 1,000 patients with an orphan disease, short term treatment of 10,000 patients with malignant glioma, or 100,000 patients with new onset Parkinson's disease. Fifth, downstream manufacturing problems, such as nanomedicine lyophilization, must be solved to ensure the nanomedicine has a commercially viable shelf-life for treatment of CNS disease in humans.

## Introduction

**Figure 1:**
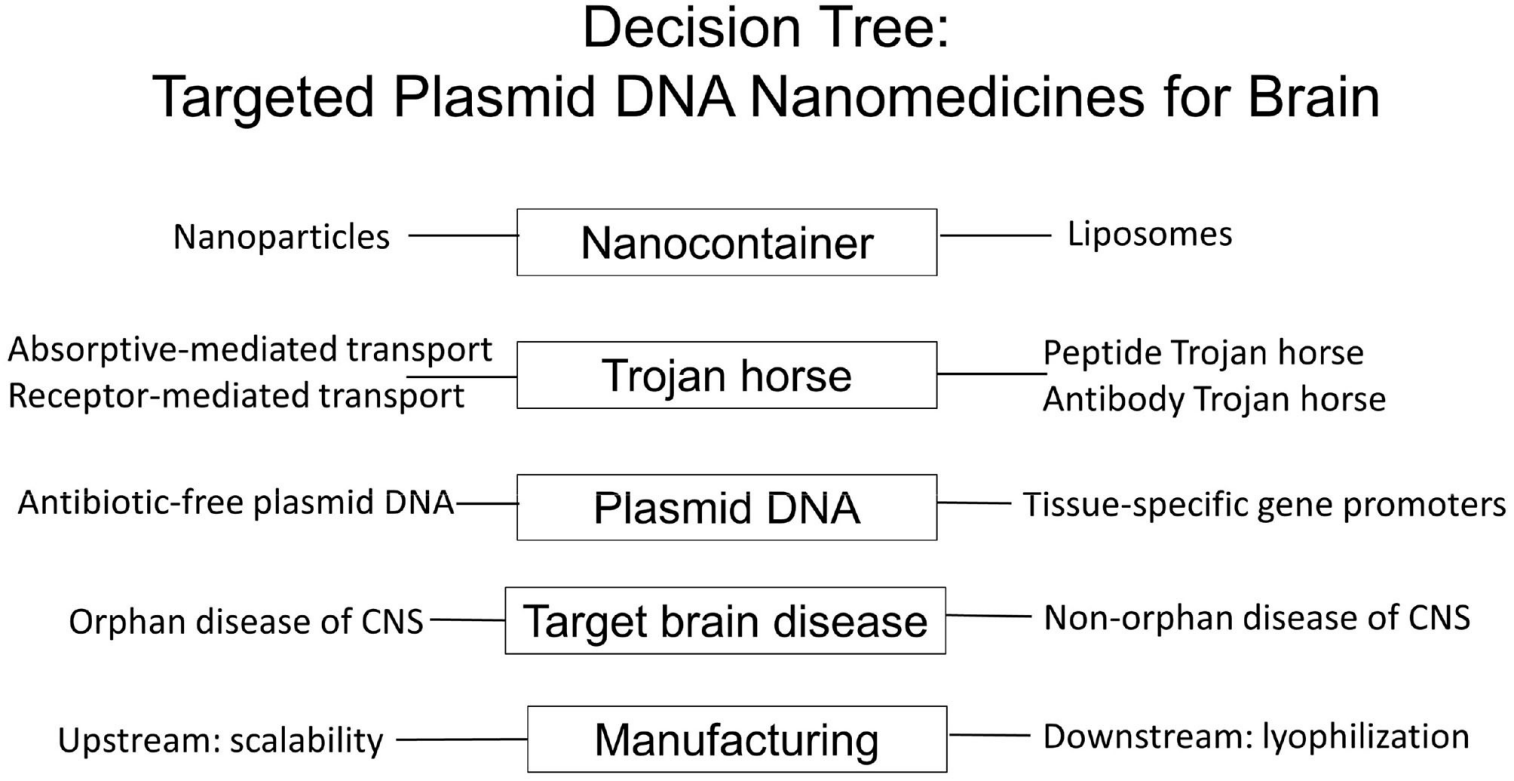
Decision tree in the formulation of BBB-penetrating nanomedicines for brain delivery of plasmid DNA therapeutics using Trojan horse liposomes for non-viral gene therapy of the brain. The variables to be considered group into at least five major areas: the type of nanocontainer, the type of BBB Trojan horse, the engineering of the antibiotic resistance free plasmid DNA with tissue specific promoters, the target brain disease, and both upstream and downstream components of the nanomedicine manufacturing, which can meet market demand of drug product for the target disease.

There are multiple considerations in the design of targeted nanomedicines for brain disease, which both encapsulate plasmid DNA encoding the therapeutic gene, and cross the blood-brain barrier (BBB), and these are outlined in Figure 1.

- **Nanocontainer**. The type of nanocontainer that encapsulates the plasmid DNA must be selected from the array of available nanocontainers, including nanoparticles or liposomes. The nanocontainer needs to be targeted to brain by attachment of a molecular Trojan horse to the surface of the nanocontainer.
- **Trojan horse**. Molecular Trojan horses are selected that enable delivery of the nanocontainer across the BBB via either absorptive-mediated transcytosis (AMT) or receptor-mediated transcytosis (RMT), and the Trojan horse may be either a peptide or a receptor-specific monoclonal antibody (MAb), which engages the targeted AMT, or RMT system on the brain capillary endothelium, which forms the BBB *in vivo*. The efficacy of BBB Trojan horses may be first investigated with *in vitro* BBB models in tissue culture, but such cell culture studies need to be validated with *in vivo* experiments that demonstrate delivery to brain. The *in vivo* validation of the BBB Trojan horse is required, because of the limitations of *in vitro* BBB models. Such models are at least 100-fold leaky compared to the BBB *in vivo*, and there is marked down-regulation of BBB tissue-specific gene expression when brain endothelial cells are grown in cell culture (1). A given RMT system may be expressed at the BBB *in vivo*, but is down-regulated in cell culture. Conversely, a BBB RMT system may be up-regulated in tissue culture, with minimal, if any, expression at the BBB *in vivo*. Owing to the leakiness of the *in vitro* BBB, compared to the BBB *in vivo*, certain Trojan horse candidates may appear promising on the basis of transport across *in vitro* BBB models, but are found to have minimal penetration of the BBB *in vivo*. It is also necessary to confirm that the receptor being targeted by the BBB Trojan horse is, in fact, expressed on the luminal membrane of the brain capillary endothelium. Certain receptors believed to be expressed on the endothelium are actually expressed on pericytes or astrocytes, which are situated beyond the BBB, and not accessible to a blood-borne Trojan horse. Without endothelial expression of the target receptor, the Trojan horse cannot trigger transport across the BBB. The *in vivo* methods used to characterize a new BBB Trojan horse must be critically evaluated, and these considerations include an assessment of the brain blood volume, the limitations of using drug entry into cerebrospinal fluid (CSF) as an index of BBB penetration, the insensitivity of immunohistochemistry as a measure of Trojan horse penetration into brain parenchyma, the use of radio-isotopic methods for measurement of Trojan horse delivery across the BBB, and the ways in which histochemistry of brain can be used to validate BBB passage of the plasmid DNA.
- **Plasmid DNA**. The Trojan horse-targeted nanocontainer encapsulates the non-viral plasmid DNA encoding the therapeutic gene. This plasmid DNA is genetically engineered with tissue-specific gene promoters upstream of the therapeutic gene. Such promoters ensure high expression in brain with minimal off-target expression. For human therapeutics, the plasmid DNA must also be genetically engineered without antibiotic resistance genes, as mandated by the FDA. The classic method of transformed bacterial clone selection is ampicillin resistance conferred by inclusion of the *ampR* gene, which encodes bacterial beta lactamase, in the plasmid DNA vector backbone. Selection of bacterial clones with antibiotics such as ampicillin must be replaced by antibiotic-free selection methods for production of plasmid DNA to be administered to humans.
- **Target brain disease**. The plasmid DNA incorporates a therapeutic gene tailored to a target disease of brain, including both orphan disease, such as genetic disease, and non-orphan diseases, such as brain cancer or Parkinson's disease (PD). Practical considerations include whether the manufacture of the nanomedicine is scalable to treat an orphan disease of brain, which may affect only 1,000 patients per year, or whether the manufacture is sufficiently scalable, so as to treat 10,000 patients per year with glioblastoma multiforme (GBM), or 100,000 patients per year with new onset PD.
- **Manufacturing**. For treatment of humans with nanomedicine therapeutics of the brain, the upstream manufacturing of the nanomedicine must be scalable so as to meet market demand for the target disease. In addition, downstream manufacturing issues must be solved, such as lyophilization of the nanomedicine, to ensure the formulation has a commercially viable shelf life.

This review will discuss each of these design elements of a brain nanomedicine, which are outlined in Figure 1. Initially, viral gene therapy of the brain is discussed, which highlights the limitations of this approach to gene therapy of the brain. The potential genotoxicity of viral gene therapy provides the rationale for the parallel development of non-viral gene therapy of brain using targeted nanomedicines that cross the human BBB.

## Viral Gene Therapy of the Brain

There are two approaches to viral gene therapy of the brain: (a) intravenous administration of stem cells permanently transfected with lentivirus encoding the therapeutic gene, and (b) intravenous administration of certain serotypes of adeno-associated virus (AAV), e.g., AAV9, which cross the BBB following intravenous administration.

### Lentiviral Transfected Hematopoietic Stem Cells

Clinical trials are ongoing for the treatment of lysosomal storage diseases that affect the CNS with human hematopoietic stem cells that are permanently transfected with lentivirus encoding the missing lysosomal enzyme (2). Retroviruses permanently integrate into the human genome, which can cause cancer (3). To minimize the risk of retrovirus-induced cancer, the FDA has set a limit of <5 for the vector copy number (VCN), or number of lentiviral genomes introduced into the stem cell (4). However, it is not possible to produce an increase in lysosomal enzyme activity in the brain of mice treated with this therapy unless the VCN > 10, and a VCN < 5 produces no therapeutic effect in brain (5). The limiting factor with this approach to brain gene therapy is the poor transport of stem cells across the BBB. Following the intravenous (IV) administration of stem cells in mice, the only region of the brain that harbors the stem cells is the meninges on the surface of the brain, where there is no BBB (6). Owing to the lack of stem cell transport across the BBB, no stem cells are found in the parenchyma of brain (6). Additional evidence that stem cells do not cross the BBB was demonstrated by measurement of the retroviral genome in brain by PCR following the IV administration of lentiviral-transduced stem cells (7). The viral genome in brain was detected at background levels, and several log orders lower than in peripheral organs (7). In summary, the lentiviral/stem cell approach to gene therapy of the brain has a narrow therapeutic index, as the VCN needed to treat the brain exceeds the FDA VCN limit of 5 allowed for human use. This narrow therapeutic index is caused by the minimal, if any, BBB transport of stem cells.

### AAV Gene Therapy of the Brain

The AAV9 serotype undergoes transport across the BBB following an IV administration. The transport is relatively inefficient, and the number of brain cells transduced is <30% following the IV injection of a high dose, 2–4 × 10^13^ vector genomes (vg)/kg, of a self-complementary (sc) form of AAV9 (8, 9). The size of the therapeutic expression cassette that can be inserted in the scAAV genome is small <2.3 kb, whereas the single stranded (ss) form of AAV can accommodate an expression cassette <4.7 kb (9). However, the transduction of brain cells by intravenous ssAAV is much less than with scAAV (9). In the 6–8 weeks old mouse, an IV injection of 4 × 10^13^ vg/kg of ssAAV9 transduces 2% of neurons and 6% of astrocytes, whereas the same dose of scAAV9 transduces 12% of neurons and 45% of astrocytes (9). Given a comparable number of neurons and astrocytes in brain, the fraction of total brain cells transduced is 4 and 28% with ssAAV and scAAV, respectively. The first FDA approved AAV gene therapy for the brain is Zolgensma® (10), which is a scAAV9 encoding the human spinal muscular atrophy (SMN)-1 gene. Zolgensma is approved for infantile spinal muscular atrophy (SMA) at an IV injection dose (ID) of 2 × 10^14^ vg/kg. For a 10 kg child, this ID represents a total of two quadrillion AAV particles in a single dose. The high dose is required owing to the inefficient transport of AAV9 across the BBB. This therapy may prove to have a narrow therapeutic index. AAV is known to permanently integrate into the human genome, particularly in the liver (11). The IV administration of 10^14^ vg/kg of AAV in newborn mice produces a >70% incidence of liver cancer later in life (12). An additional problem with AAV gene therapy is the immune response formed against the viral capsid protein after just a single administration (13). Moreover, the immune response against AAV is also directed against the protein product of the therapeutic gene. Primates administered AAV encoding the lysosomal enzyme, N-acetyl-alpha-glucosaminidase (NAGLU), produce neutralizing antibodies against the NAGLU enzyme (14). The strong immune response formed against a single injection of AAV precludes the administration of a second dose of the virus, which is why AAV is only approved for a single treatment. Similar to lentiviral gene therapy of the brain, intravascular AAV gene therapy of the brain has a narrow therapeutic index. The dose of scAAV9 administered to humans, 2 × 10^14^ vg/kg (10), is the same dose that causes a high incidence of delayed onset of hepatocellular carcinoma in mice (12).

The potential genotoxicity of viral gene therapy of the brain provides the basis for a parallel effort in the development of non-viral gene therapy of the brain using targeted nanomedicines. Such nanomedicines must be engineered so as to enable passage of plasmid DNA through the BBB and into brain cells. This review will focus on the use of molecular Trojan horses to facilitate transport of plasmid DNA nanomedicines across the BBB.

## Nanocontainers For Plasmid DNA Delivery to Brain

### Pegylated Immunonanoparticles

**Figure 2:**
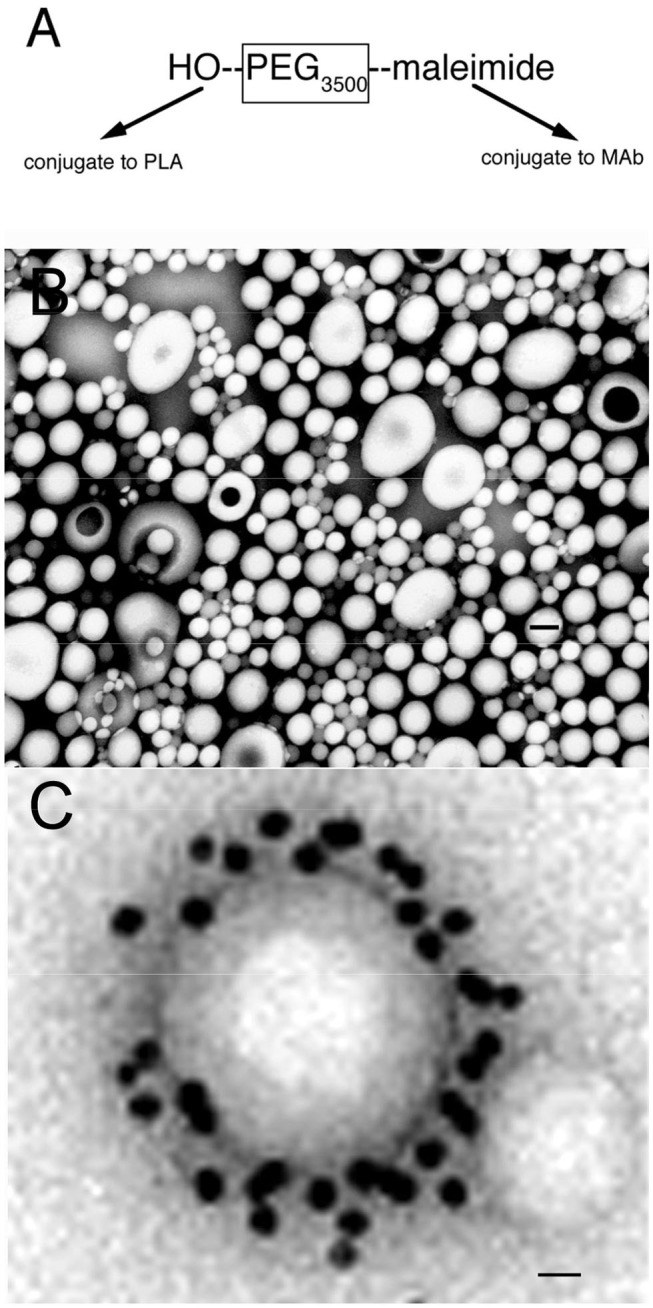
**(A)** Bi-functional 3500 Da polyethyleneglycol (PEG_3500_) containing a free hydroxyl group at one terminus, for conjugation to the poly (L-lactide) (PLA), and a maleimide moiety at the other terminus, for conjugation to a thiolated receptor specific monoclonal antibody (MAb). **(B)** Transmission electron microscopy of pegylated nanoparticles. Magnification bar = 120 nm. **(C)** Electron micrograph of pegylated immunonanoparticle mixed with a secondary antibody directed against the MAb conjugated to the surface of the nanoparticle, where the secondary antibody is a conjugate of 10 nm gold. Magnification bar = 15 nm. Reprinted by permission of Olivier et al. (20).

Nanoparticles are formed from degradable biopolymers such as poly (L-lactide) (PLA), poly (DL-lactide, coglycolide) (PLGA), or polybutylcyanoacrylate (PBCA) (15). During production, the nanoparticles are stabilized with an amphiphilic surfactant, such as polyvinyl alcohol (PVA) or pluronic copolymers (16), polysorbates, such as Tween-80 (17), or bile acids, such as cholic acid (18). Nanoparticles are rapidly cleared from blood following IV administration, owing largely to uptake by liver. This hepatic uptake, and the rapid removal from plasma, was reduced by conjugation of 2000 Da polyethyleneglycol (PEG^2000^) to the surface of the nanoparticle (19). Receptor targeting of pegylated nanoparticles was enabled with the conjugation of a receptor specific MAb to the tips of the PEG strands and the production of pegylated immunonanoparticles (PIN). This was possible by synthesis of hydroxy-polyethyleneglycol^3500^-maleimide (Figure 2A). Methoxy PEG^2600^-poly(lactic acid)^40000^, designated methoxyPEG^2600^-PLA^40000^ copolymer, and a maleimide-PEG^3500^-PLA^40000^ copolymer, were synthesized as described previously (20). An emulsion/solvent evaporation technique (21) was used to produce pegylated PLA nanoparticles from a blend of methoxyPEG^2600^-PLA^40000^ and maleimide-PEG^3500^-PLA^40000^ (20). The emulsion was prepared by sonication of the copolymers in a mix of water and dichloromethane with 1% sodium cholate as a surfactant followed by removal of the dichloromethane by rotary evaporation, and the nanoparticles were collected by centrifugation at 45,000 g. The diameter of the pegylated nanoparticles was measured by dynamic light scattering and was 121 ± 5 nm. The pegylated nanoparticles were examined by electron microscopy, and the nanoparticles were generally ~100 nm, although some were as large as 300 nm (Figure 2B). In parallel to production of the pegylated nanoparticles, the OX26 mouse MAb against the rat transferrin receptor (TfR) was thiolated with Traut's reagent, and the thiolated MAb was conjugated to the maleimide moieties on the tips of the PEG strands on the surface of the nanoparticle. These PINs were purified by gel filtration to remove the unconjugated TfRMAb. The number of TfRMAb molecules conjugated per nanoparticle was 67 ± 5 (20). The conjugation of the TfRMAb to the PIN surface was demonstrated by electron microscopy following mixture of the PIN and a conjugate of an anti-mouse IgG and 10 nm gold (Figure 2C). The different methods of conjugation of a nanoparticle with a targeting MAb (22, 23) or a targeting ligand have been reviewed (24, 25). While PINs are an attractive approach to targeting non-DNA drugs to brain, this formulation was considered problematic for encapsulation of super-coiled plasmid DNA. The sonication required to prepare the emulsion is known to nick super-coiled plasmid DNA (26, 27), which can cause a decrease in transfection potency of the plasmid DNA. In addition, the surfactant, which is used to reduce aggregation of the nanoparticle (28), may have separate pharmaceutical effects (16, 29), including enhancement of BBB transport (17, 30). Therefore, pegylated immunoliposomes, also called Trojan horse liposomes (THLs), were developed as the nanocontainer for plasmid DNA delivery to brain. THLs are distinct from cationic liposomes, which are a form of cationic polyplex with plasmid DNA.

### Cationic Polyplexes

Cationic polyplexes are a mixture of a cationic polymer and anionic plasmid DNA. If the cationic polymer is a lipid, then the polyplex is often referred to as cationic liposomes. With cationic liposomes, the plasmid DNA is not encapsulated in the interior of the liposome, and is susceptible to degradation by nuclease (31). Plasmid DNA polyplexes form nanoparticles in water, but aggregate in physiologic saline to form micron size structures (32), which triggers the phagocytic uptake in cultured cells that enhances transfection *in vitro* (33). However, *in vivo*, this aggregation in saline causes embolization of the cationic/DNA polyplex in the pulmonary capillary bed (34). Cationic DNA polyplexes do not cross the BBB and must be administered to brain via intra-cerebral injection (35). To enable the production of nuclease-resistant nanomedicines that cross the BBB, Trojan horse liposomes were developed.

### Magnetic Nanoparticles

Iron oxide magnetic nanoparticles (MNP) are generated from iron (Fe) at high temperatures (36), and can be localized in the brain vasculature by placing the subject in an external magnetic field (EMF). So as to improve brain penetration of MNPs, these formulations were prepared with a surfactant, Tween 80 (36), and the surfactant enhanced brain uptake of the MNPs similar to non-magnetic nanoparticles. MNPs have been prepared with either gold (Au) or Au/Fe mixtures. Such nanoparticles have a diameter of only 2.5 nm and are believed to traverse the BBB via calcium, potassium, or sodium channels (37). However, the pore size of a calcium or potassium channel is only 9–15 Å (0.9–1.5 nm) (38–40). While the gold nanoparticle may have a diameter of only 2.5 nm (37), the diameter of drug conjugated gold nanoparticles is larger. Short interfering RNA (siRNA) against the Bcl2L12 oncogene was complexed to gold conjugated nanoparticles to produce structures with a diameter of 19-34 nm (41). It is not clear how MNPs of this size can traverse the small pore of cation channels. MNPs have been proposed for delivery of plasmid DNA, where the MNP, the plasmid DNA, and a cationic polymer, such as polyethylenimine, are mixed (42). As discussed above, such cationic polyplexes aggregate in physiological saline. The diameter of the DNA/MNP formulation was 150–200 nm in water, but the complexes aggregated in physiologic medium causing the diameters of the MNPs to exceed 1,000 nm (42). This aggregation promotes complex uptake in cultured cells (33), but restricts brain uptake *in vivo* similar to other cationic polyplexes, owing to entrapment of the aggregates in the lung (34). In the case of MNP/DNA cationic polyplexes, there is gene expression in a variety of culture cell lines, but *in vivo* gene expression in lung is 10-fold higher than in other peripheral organs with no gene expression in brain (43).

### Trojan Horse Liposomes

Trojan horse liposomes (THL), also called pegylated immunoliposomes, are formed by encapsulation of plasmid DNA in the interior of pegylated liposomes that have a net anionic charge, and the tips of 1–2% of the surface PEG strands are conjugated with a MAb that targets an endogenous receptor expressed on both the BBB endothelium and on brain cells (44). The receptor specific MAb acts as a molecular Trojan horse by binding the endothelial receptor, and this binding triggers transport across the BBB, and then by binding the receptor on brain cells, which triggers endocytosis into brain cells beyond the BBB. THLs are similar to stabilized plasmid lipid particles (SPLP), which are pegylated liposomes that encapsulate plasmid DNA in the interior of the liposome (45), except SPLPs have no surface targeting ligand. Pegylated liposomes without a targeting ligand, such as SPLPs, do not cross the BBB *in vivo* (46).

**Figure 3:**
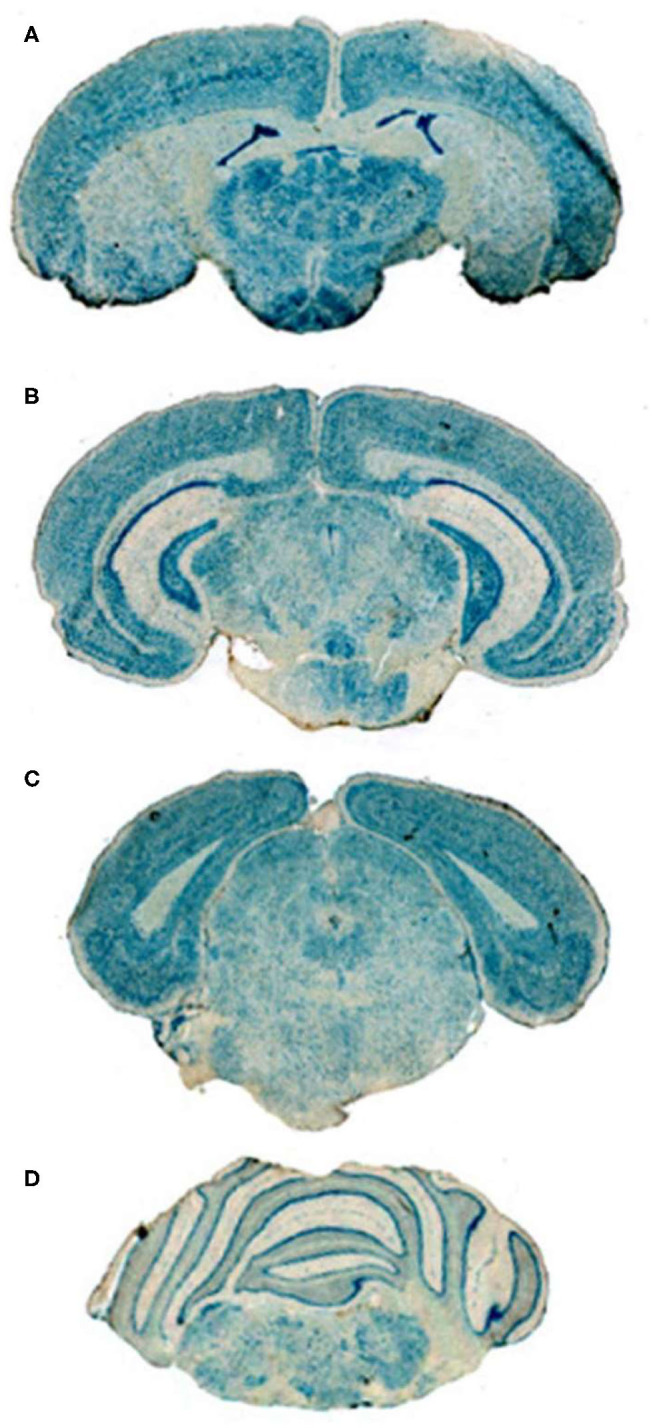
X-gal histochemistry of mouse brain removed 48 h after IV administration of THLs targeted with the 8D3 MAb against the mouse TfR, and encapsulating a LacZ β-galactosidase expression plasmid DNA. The dose of THL encapsulated plasmid DNA is 5 ug/mouse. **(A)** Rostral diencephalon, **(B)** rostral mesencephalon, **(C)** caudal mesencephalon, **(D)** rostral cerebellum. The study shows global expression of the transgene throughout the brain following IV administration of THLs. Reprinted with permission of Zhu et al. (47).

Conjugation of a MAb Trojan horse to the surface of THLs enables this nanocontainer to cross the BBB and enter brain cells following IV administration, owing to engagement of receptors on the BBB by the MAb on the THL surface. The transfection of brain *in vivo* with THLs in illustrated in Figure 3, which is X-gal histochemistry of mouse brain removed 2 days after the IV injection of a TfRMAb-targeted THL encapsulated with a LacZ expression plasmid DNA encoding β-galactosidase (47). Expression of the LacZ gene is found throughout the mouse brain including the rostral diencephalon, rostral mesencephalon, caudal mesencephalon, and rostral cerebellum (Figures 3A–D, respectively). If the TfRMAb on the THL is replaced by a non-specific IgG, then no LacZ gene expression is observed in mouse brain (48). Light microscopy of the mouse brain shown in Figure 3 confirms LacZ expression in neurons in brain (47). The brain expression of the LacZ gene following IV administration of THLs has also been reported for rats using the OX26 TfRMAb specific for the rat TfR (49), and in Rhesus monkeys (50), using a MAb against the human insulin receptor (HIR), where this HIRMAb cross reacted with the primate insulin receptor (51). In addition to brain cells, the transgene is also expressed in the choroid plexus epithelium and the microvascular endothelium in monkey brain following the IV administration of THLs encapsulating the β-galactosidase expression plasmid DNA (50).

A luciferase reporter gene plasmid DNA was delivered to the brain of Rhesus monkeys following the IV administration of HIRMAb-targeted THLs (50). Peak brain luciferase enzyme activity was 9 pg/mg brain protein following the IV injection of a dose of THL encapsulated plasmid DNA of 12 μg/kg. Based on the size of this double stranded plasmid DNA, 10.6 kb, the injection dose of 12 μg/kg of THL encapsulated plasmid DNA is equivalent to 10^12^ vector genomes (vg)/kg, where 1 vg equals 1 plasmid DNA molecule.

THLs were produced with the thin film/extrusion method with the following phospholipids: 1-palmitoyl-2-oleoyl- *sn*-glycerol -3-phosphocholine (POPC); dimethyldioctadecylammonium bromide (DDAB); DSPE-PEG^2000^-MAL, where DSPE = 1,2-distearoyl-sn- glycero-3- phosphorylethanolamine, PEG^2000^ = 2000 Da polyethyleneglycol, and MAL = maleimide; and DSPE-PEG^2000^, in a molar ratio of 9.3:0.3:0.1:0.3 of POPC:DDAB:DSPE-PEG^2000^-MAL:DSPE-PEG^2000^ (44, 52). Although DDAB is a cationic lipid, the DSPE-PEG^2000^, an anionic lipid, is present in a molar excess over the cationic lipid. Following evaporation to a thin film, the phospholipids are hydrated, sonicated, and mixed with super-coiled plasmid DNA. Following several freeze-thaw cycles, small liposome vesicles were formed by extrusion through polycarbonate filters of 400, 200, and 100 nm pore size. The plasmid DNA not encapsulated in the interior of the liposome was removed by treatment with endonuclease I and exonuclease III. Plasmid DNA encapsulated in the interior of the liposomes is resistant to external nucleases. In parallel, the targeting MAb was thiolated with Traut's reagent, and the thiolated MAb was conjugated to the MAL moiety at the tip of the PEG strands. The unreacted MAb and degraded DNA were removed from the THL preparation by Sepharose CL4B gel filtration chromatography (52). Several modifications to the original method have been recently described (53, 54). First, the amount of phospholipid used to encapsulate 400 μg DNA was reduced 4-fold from 40 μmol to 10 μmol, as this was found to reduce the number of DNA-free or empty liposomes to <10% of total. Second, the plasmid DNA was added to the hydrated lipids in 40% ethanol, as this increases the encapsulation of the DNA in the interior of the liposomes (55, 56). Third, a small amount of cholesterol, 6% of total phospholipid, is added as this amount of cholesterol stabilizes membranes (57). Fourth, the THL production may be scaled up from the 10 μmol phospholipid stage, which uses the hand-held LF-1 extruder (52), to the 50 μmol phospholipid stage, which uses the pressure driven LF-50 extruder (53). The optimization of the THL manufacturing process, including use of a scalable ethanol dilution manufacturing, as well as a THL lyophilization process, are described below in the Manufacturing section. Fifth, a recombinant form of a TfRMAb (54) or a HIRMAb (53) was incorporated into the THLs.

#### Dual Receptor Targeting

THLs may be targeted to two different receptors in brain by conjugation of two different MAb molecules to the THL surface. In an intracranial human brain cancer model in scid mice (58, 59), the THL was delivered across the tumor capillary, which originated from mouse brain, with the rat 8D3 MAb to the mouse TfR. However, this MAb does not recognize the human TfR on the human glioma cancer cell. Therefore, the THL was also conjugated with the HIRMAb. This antibody does not recognize the mouse insulin receptor (60), but does bind to the HIR on the glioma cell (59). Dual antibody THLs have also been prepared with the mouse OX26 MAb against the rat TfR, to target the BBB, and a second MAb against α-synuclein, to target this protein in Parkinson's disease (61). Dual targeting in brain has also been tested with the OX26 MAb to target the BBB, and the scorpion-derived chlorotoxin (CTX) neuropeptide, to target glioma cells (62). Glial cells may also be targeted with a MAb against glial fibrillary acidic protein (GFAP) (63).

#### Avidin-Biotin Technology

The targeting MAb may also be attached to the THL surface with avidin-biotin technology. The DSPE-PEG-MAL lipid is replaced by DSPE-PEG-biotin (64). The OX26 TfRMAb was then attached to streptavidin (SA) via a thiol-ether linker to produce the TfRMAb/SA conjugate, which bound the biotin groups on the surface of the THL (64). Alternatively, the rat RI7-217 MAb against the mouse TfR was biotinylated, in parallel with production of liposomes with phospholipid-PEG-biotin. The mixture of the liposome-biotin, the biotinylated TfRMAb, and SA resulted in binding of the liposome to the TfRMAb via the SA bridge (65). The SA bridge method was also used by Loureiro et al. (61).

#### Post-Insertion Liposomes

The targeting MAb may be incorporated into the surface of the liposome with the post-insertion method (66). In this approach, the liposome is prepared without a surface MAL moiety. In parallel, micelles are produced by heating at 65°C the DSPE-PEG and DSPE-PEG-MAL, followed by conjugation of the thiolated MAb to the MAL moiety on the micelles. The micelles are then incorporated into the liposome surface by incubation of the liposomes and the micelles at 60°C for 1 h (66). THLs were recently produced with the post-insertion method and the OX26 TfRMAb (67).

#### Encapsulation of Large Size Plasmid DNA

Plasmid DNA as large as 22 kb have been incorporated into THLs, and such large plasmids produced *in vivo* gene expression in brain following IV administration (68). In contrast, there is a size limitation of transgene expression cassettes which can be incorporated into AAV viral vector, e.g., 2.3 kb for scAAV and 4.7 kb for ssAAV (9). The ability to encapsulate large size plasmid DNA in THLs allows for the use of tissue-specific gene promoters, including chromosomal derived DNA, which may be several kb in size, and the engineering of plasmid DNA with tissue-specific promoters for encapsulation in THLs is described below.

With respect to the Trojan horse that is incorporated in the THL, there is a large array of either peptide-based or IgG-based Trojan horses that have been used. Peptide-based Trojan horses may cross the BBB via either absorptive-mediated transcytosis (AMT) or receptor-mediated transcytosis (RMT). Trojan horses that cross the BBB via RMT may target any number of receptors on the BBB apart from the TfR or the insulin receptor. The mechanism of BBB transport (AMT, RMT), the type of Trojan horse (peptide, IgG), the localization in brain of the receptor targeted by the Trojan horse, and methods used to confirm BBB delivery *in vivo* of Trojan horses, are reviewed below.

## Blood-Brain Barrier Trojan Horses

### Absorptive-Mediated Transcytosis Through the Blood-Brain Barrier

Absorptive-mediated transcytosis (AMT) through the BBB is mediated via either electrostatic or carbohydrate interactions between the ligand and the endothelial surface. The electrostatic AMT occurs following the interactions of a cationic ligand and anionic charges, which are found on the endothelial plasma membrane (69). The carbohydrate AMT is triggered by the binding of a lectin to carbohydrate groups on the endothelial plasma membrane.

#### AMT of Cationic Trojan Horses

Cationic ligands that trigger AMT include cationized albumin (70), histone (71), and cell penetrating peptides (CPP), such as the cationic peptides, tat, polylysine, polyarginine, or the 19 amino acid polycationic SynB1 peptide (72). These cationic ligands are rapidly taken up by cells in tissue culture. However, the brain uptake of CPPs *in vivo* is minimal. At 60 min following the IV injection of tat, polyarginine, or SynB1, the brain uptake in the mouse is at the background level of 0.1% injected dose (ID)/gram (72). Fusion of the tat peptide, GRKKRRQRRRPPQ, a 12 amino acid cationic sequence derived from the HIV-1 viral tat protein, to a model lysosomal enzyme, β-glucuronidase (GUSB), resulted in the formation of a tat-GUSB fusion protein. IV administration to mice showed high uptake in liver and spleen but a background level of brain uptake of the tat-GUSB fusion protein (73). The minimal brain uptake of the tat-GUSB fusion protein (73) is due to the very low uptake of a CPP, such as the tat peptide, by the brain *in vivo* (72).

AMT of IgG was examined following the cationization of the IgG molecule, and the cationized IgG was rapidly taken up by isolated brain microvessels *in vitro* via a process that was saturable but with low affinity and an ED50 of 0.90 ± 0.37 μM (74). However, cationized Trojan horses, such as cationized albumin or cationized IgG, are not as robust BBB delivery systems as the MAb Trojan horses that target BBB RMT systems. First, the binding of the cationized ligand to the BBB is weak with an ED50 in the μM range (74, 75), whereas RMT ligands are generally high affinity with ED50 values in the low nM range, as discussed below. Second, cationic Trojan horses are sequestered within the intra-endothelial compartment of the brain microvasculature. This was demonstrated by carotid arterial infusion coupled with the capillary depletion method, which showed predominant distribution of cationized ligands in the vascular pellet (71). In contrast, either Tf, or a TfRMAb, is minimally distributed in the vascular pellet with predominant distribution in the post-vascular supernatant, which is indicative of transcytosis through the endothelial barrier (76). Third, cationic ligands cause toxicity *in vivo*. Histone, a naturally occurring 14 kDa cationic protein, causes BBB disruption following carotid arterial infusion (71). The administration of 15–40 mg/kg of naturally occurring cationic proteins such as histone or protamine causes protein extravasation *in vivo* (77, 78). The IV administration of amino acids 130–149 of apoE, which is a cationic peptide, to mice causes injection related reactions and death (79).

#### AMT of Lectin Trojan Horses

Lectins are plant proteins, and some lectins may trigger AMT across the BBB. Wheat germ agglutinin (WGA) is a lectin, which binds to N-acetyl D-glucosamine sites on the endothelial membrane, and this binding triggers transport through the BBB, as demonstrated electron microscopically with a WGA-horseradish peroxidase (HRP) conjugate (80). Similar to the cationic ligands, WGA is sequestered within the lysosomal compartment of cells with minimal transcytosis (81). WGA administration *in vivo* stimulates an inflammatory reaction with production of multiple cytokines (81). Pegylated nanoparticles conjugated with WGA cause cytoxicity in Calu-3 cells, which was not observed with pegylated nanoparticles lacking the WGA ligand (82).

#### AMT of Glycopeptide Trojan Horses

Glycopeptides were formed by conjugation of a D-glucose moiety to an oligopeptide, and were initially believed to traverse the BBB via the GLUT1 glucose transporter, although there was no direct evidence for transport via the GLUT1 pore (83). Subsequent work showed the mechanism of transport of the glycopeptide was AMT via a novel mechanism of “membrane hopping” (84). Such a mechanism of membrane transport is reminiscent of that proposed by Trauble (85) for “molecular hitchhiking” of small molecules through biological membranes. The small molecules move through transitory holes within the phospholipid membrane, and such “holes” are caused by the kinking of membrane phospholipids (85). However, the size of the transitory holes estimated by Trauble (85) for small molecule transport would seem to be too small to accommodate the membrane hoping of the larger oligopeptide. The upper limit of size of small molecule transport through the BBB is about 400 Da, which corresponds to a molecular surface area about 100 Å^2^ (86). The transport of small molecules through the BBB is minimal when the molecular weight produces a molecular surface area >100 Å^2^ (86). The surface area of raffinose, a 504 Da trisaccharide, is 267 Å^2^ (87), which would be a lower bound for the molecular surface area of oligopeptides. Oligopeptides, even as small as the five amino acid leucine enkephalin, which has a molecular weight of 556 Da, have a molecular surface area >100 Å^2^ threshold for diffusion through the BBB.

#### AMT of Fusogenic Viral Proteins

A beta galactosidase reporter plasmid DNA was encapsulated in liposomes conjugated with the hemagglutinin virus of Japan (HVJ) envelope protein, and histochemistry of brain showed transgene expression in scattered microvessels of brain (88). The HJV, also designated the Sendai virus (SeV), is known to invade endothelial cells (89). Such fusogenic viral proteins are an alternative to cationic cell penetrating peptides (CPP), which are largely confined to the endosomal/lysosomal compartment of cells, whereas non-cationic viral fusogenic proteins (FP) undergo endosomal escape (90). Fusogenic viral envelope proteins include the influenze virus hemagglutinin, the flavivirus E protein, and the vesicular stomatitis virus G protein (91).

### Receptor-Mediated Transcytosis Through the Blood-Brain Barrier

Small molecule nutrients, hormones, or vitamins cross the BBB via carrier-mediated transport (CMT). CMT systems are transmembrane proteins that form pores or gates that open and close to allow for substrate transport through the membrane. CMT systems generally do not undergo endocytosis to mediate transport, as in the case of RMT transport. Instead, the CMT pore traverse the entire width of the membrane and acts as a gate that opens to allow entry of the small molecule substrate, and then closes as the substrate traverses the pore to be released to the opposite side of the membrane, as illustrated in the case of the GLUT1 glucose transporter (92). The CMT system generally rests within the membrane throughout the transport cycle. In contrast, RMT systems are transmembrane receptors, which first bind the ligand, and then the ligand/receptor complex is internalized into the cytoplasmic compartment, thus causing the receptor to exit the plasma membrane and move to an intracellular endosomal membrane (93). The receptor separates from the ligand and undergoes retro-endocytosis to return to the plasma membrane, or the ligand traverses the length of the cell to undergo transcytosis across the cell barrier. The very different membrane trafficking mechanisms of CMT and RMT transport have implications for BBB drug delivery. It is difficult to conjugate a drug to a CMT substrate and expect to find that the drug-substrate complex is still accepted by the CMT pore, which is generally stereospecific and characterized by strict structure-activity relationships among substrates. Conversely, it is easier to conjugate a drug to a RMT ligand, as the endocytosis mechanism will still mediate the transport of the drug/RMT ligand, providing this conjugation does not interfere with binding of the ligand to the RMT system.

#### Characterization of BBB RMT Systems

The BBB expresses several RMT systems for endogenous peptides, including insulin, transferrin (Tf), insulin-like growth factor (IGF)-1 and IGF-2, and leptin. The high affinity binding of these peptides to the respective RMT system on the human BBB has been characterized by radio-receptor assays and isolated human brain microvessels (1), and the low nM KD values of peptide/receptor binding at the human BBB range are 1.2 ± 0.5, 5.6 ± 1.4, 1.1 ± 0.1, 2.1 ± 0.4, and 5.1 ± 2.8 nM for the receptor binding of insulin, transferrin, IGF-2, IGF-1, and leptin, respectively (94). If a given RMT system is said to mediate the BBB transport of a given ligand, then several properties of this transport process should be experimentally confirmed. First, the RMT system must be expressed at the capillary endothelial cell as demonstrated microscopically with light or electron microscopic immunohistochemistry or fluorescent microscopy. Second, BBB transport of the ligand via the RMT system should be saturable. Owing to the limitations of *in vitro* BBB models (1) discussed below, the saturable transport of the RMT ligand across the BBB should be demonstrated *in vivo*, e.g., with carotid arterial infusion methods, as has been done for insulin transport (95), IGF transport (96), and transferrin transport (76). Third, the *in vitro* binding of the ligand to the receptor on isolated brain microvessels or microvessel membranes should be saturable, e.g., as demonstrated with radio-receptor assays. Fourth, the molecular weight (MW) of the saturable binding site on the BBB should be identified, e.g., with affinity cross-linking sodium dodecyl sulfate polyacrylamide gel electrophoresis (SDS-PAGE), and this MW should be comparable to the MW of the same receptor expressed in peripheral tissues.

#### Immunohistochemical Localization of Targeted Receptor at Brain Endothelium

**Figure 4:**
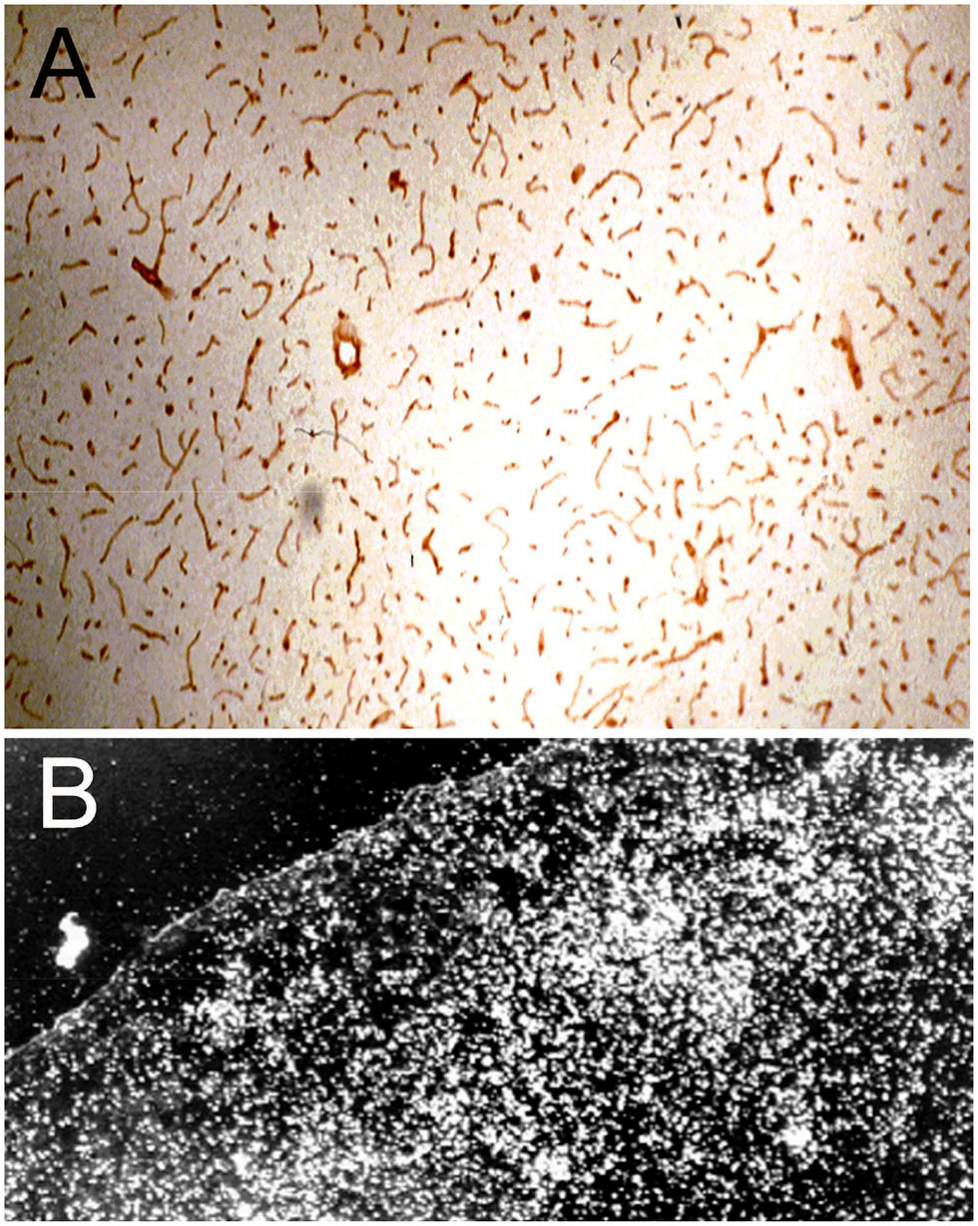
**(A)** Immunocytochemistry of rat brain with a primary rabbit antiserum against all isoforms of the human leptin receptor (LEPR). The 10 micron frozen section of brain was fixed in acetone for immune staining. Magnification = 34X. The study shows high expression of the LEPR at the brain microvessel; the continuous immune staining of the vessels indicates the cell origin of the immunoreactive LEPR is the capillary endothelium. Reproduced with permission of Boado et al. (97). **(B)** Darkfield thaw mount emulsion autoradiography of rat brain removed 5 min after an internal carotid artery infusion of [^125^I]-rat holo-transferrin (Tf); the brain was saline cleared for 30 s following the 5 min infusion of [^125^I]-Tf. The diffuse distribution of silver grains throughout the brain parenchyma indicates the Tf has rapidly transcytosed through the BBB during the 5 min arterial infusion to distribute throughout the parenchyma of brain. Reprinted with permission of Skarlatos et al. (76).

**Figure 5:**
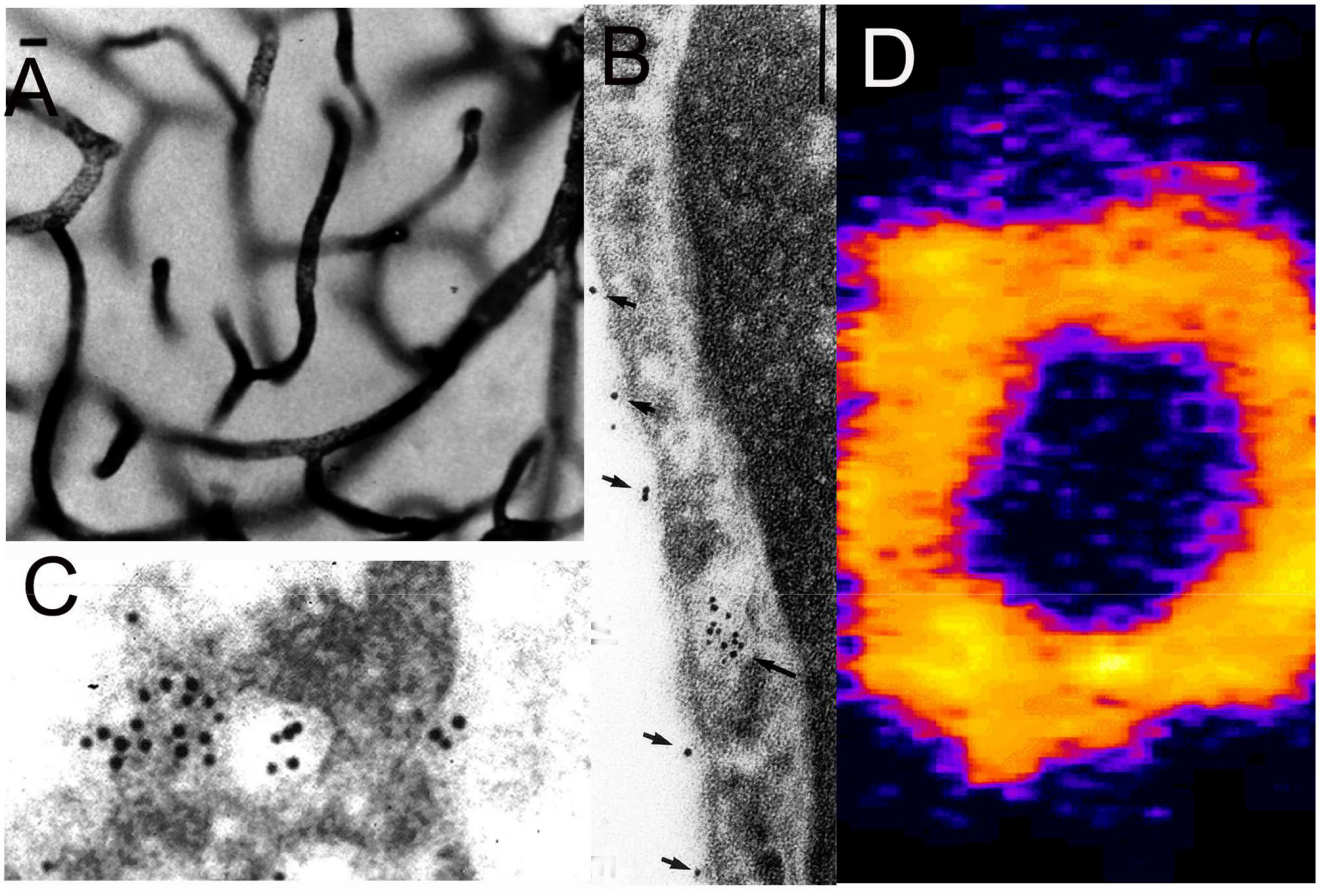
**(A)** Light microscopic silver staining of rat brain following a 10-min internal carotid artery infusion of a conjugate of 5 nm gold (Au) and the OX26 MAb against the rat TfR. Prior to perfusion fixation of rat brain with 2% glutaraldehyde, the brain vasculature was cleared with a saline infusion. Magnification bars = 10 microns in **(A)** and 100 nm in **(B)**. **(B,C)** Electron microscopy of rat brain; the OX26 MAb—gold conjugate is observed both bound to the endothelial luminal membrane (arrows, **B**), and entrapped within the intra-endothelia compartment within 100 nm endosomes (arrow in **B**). The TfRMAb-Au conjugate is observed exocytosed across the abluminal membrane into the brain interstitium (**C**). **(A–C)** are reproduced with permission from Bickel et al. (109). **(D)** Confocal microscopy of freshly isolated unfixed rat brain capillaries shows labeling of the BBB TfR on both the luminal and abluminal endothelial plasma membranes by the rhodamine labeled OX26 TfRMAb conjugated THLs. Endothelial membranes are purple and intra-endothelial cytoplasm is yellow. Panel D is reproduced with permission of Huwyler and Pardridge (110).

The immunohistochemical demonstration of the leptin receptor (LEPR) on the brain microvasculature in the rat is illustrated in Figure 4A (97). The continuous immune staining of the brain microvessel is indicative of an endothelial origin of the LEPR, as receptor expression in pericytes or astrocyte foot processes would produce a discontinuous pattern of microvascular staining. The LEPR was similarly shown to be expressed at the BBB in human brain (97). The endothelial expression of the insulin receptor (IR) was demonstrated by immunohistochemistry of the primate brain (51). An excellent immunohistochemical demonstration of continuous immune labeling of the capillary endothelium in mouse brain with an antibody against the IR is shown by Kurata et al. (98). Confocal microscopic demonstration of the expression of the TfR at both luminal and abluminal endothelial membranes is shown in Figure 5D.

#### Saturation of RMT Transport *in vivo*

The saturable transport of insulin across the BBB in the rabbit was demonstrated by carotid arterial infusion of [^125^I]-insulin and light microscopic emulsion autoradiography (95). These methods were replicated for [^125^I]rat holo-transferrin (Tf), where the labeled Tf was infused in the carotid artery of rats for 5 min followed by 30 s of saline clearance of the brain blood volume (76). The brain was then removed and frozen brain sections were applied to emulsion coated slides for thaw-mount emulsion autoradiography. Darkfield illumination of the slides shows the Tf has rapidly transcytosed through the BBB followed by entry into brain parenchyma throughout the brain during the 5 min infusion period. The carotid arterial infusion of [^125^I]-Tf was repeated with the addition of 10% rat serum to the infusate. Serum contains ~25 μM of Tf, and 10% serum carries 2,500 nM Tf. The KD of Tf binding to the BBB TfR is 5 nM (99). Therefore, the endogenous Tf in 10% rat serum causes >99% saturation of the BBB TfR. The addition of the rat serum to the infusate reduced the number of silver grains in brain parenchyma by >99%, which confirms the saturation of the BBB transport of Tf (76).

#### Saturation of Receptor Binding *in vitro* and Determination of Receptor Molecular Weight

The saturable binding of certain endogenous peptides to the cognate RMT systems at the BBB was demonstrated by radio-receptor assays and isolated human brain capillaries (1). However, the peptide could be binding to a receptor not related to its cognate receptor. Binding of the ligand to the cognate receptor was demonstrated for insulin binding to the human BBB IR (100), and for IGF-1 and IGF-2 to the human BBB IGF receptor (IGFR) (101). [^125^I]-insulin was bound to human BBB membranes and affinity cross-linked to the binding site with disuccinimidyl suberate, followed by SDS-PAGE and autoradiography. The MW of the only saturable binding site on the BBB for insulin was 127 kDa, which is identical to the MW of the alpha subunit of the IR in peripheral tissues (100). Similarly, affinity cross-linking of [^125^I]-IGF1 or [^125^I]-IGF2 to human brain microvessel membranes showed the only saturable binding site had a MW of 141 kDa for either peptide (101), which is identical to the size of the alpha subunit of the IGF1R in peripheral tissues. Both IGF1 and IGF2 produce high affinity binding to the IGF1R with KD values of 0.3 and 2.3 nM, respectively (102, 103). IGF2 also binds with high affinity to the cation independent mannose 6-phosphate receptor (CI-M6PR), and the MW of this receptor is 250 kDa (104). However, affinity cross-linking studies show the CI-M6PR is not expressed at the human BBB (101), which is why lysosomal enzymes do not cross the BBB, despite the high content of mannose-6 phosphate on these enzymes (105).

#### BBB Transferrin Receptor: transcytosis vs. endocytosis

The finding of rapid transport of Tf through the BBB *in vivo* (Figure 4B) indicates the BBB TfR mediates the transcytosis of Tf through the BBB, rather than simply the endocytosis of blood borne holo-Tf into the endothelial cytoplasm followed by the retro-endocytosis of apo-Tf back to blood. Early support for the retro-endocytosis model of BBB Tf transport came from *in vivo* studies on the brain uptake of Tf labeled with both [^125^I] and [^59^Fe]. Following the IV injection of Tf co-labeled with [^125^I] and [^59^Fe] in rats, brain [^59^Fe] content exceeded that of the [^125^I]-Tf, which suggested Tf released the iron within the capillary endothelium followed by reverse endocytosis of apo-Tf to blood (106). However, these data are also consistent with the transcytosis model of BBB transport of Tf. The greater brain uptake of [^59^Fe] over [^125^I]-Tf is consistent with rapid penetration of holo-Tf through the BBB, as demonstrated in Figure 4B, followed by release of iron in brain cells and rapid reverse transcytosis of apo-Tf back to blood. The rapid efflux from brain to blood of apo-Tf was demonstrated with the Brain Efflux Index method (107). Early support for the retro-endocytosis model also came from a pre-embedding immunocytochemical study of the TfR at the brain capillary; the TfR was localized only to the luminal endothelial membrane, and was not found on the abluminal endothelial membrane (108). However, it is known that pre-embedding immunocytochemistry cannot detect abluminal receptors in brain endothelium, as these are not exposed with this methodology (69). The confocal microscopy of freshly isolated unfixed rat brain capillaries shows expression of the TfR on both luminal and abluminal membranes (Figure 5D).

Support for the BBB TfR transcytosis model was produced with an electron microscopic study of rat brain following a 10 min carotid arterial infusion of a conjugate of 5 nm gold (Au) and the OX26 TfRMAb (109). The TfRMAb-Au conjugate was infused in the carotid artery for 10 min followed by a 60 s saline flush of the brain blood volume, followed by perfusion fixation with 2% glutaraldehyde. The distribution in brain of the TfRMAb-Au was assessed at the light microscopic level with immunogold-silver staining (Figure 5A), and at the electron microscopic level (Figures 5B,C). At the electron microscopic level, the TfRMAb-Au conjugate is visible within ~100 nm endosomes within the endothelial intracellular compartment (Figure 5B), and the TfRMAb-Au conjugate is shown to undergo exocytosis across the abluminal endothelial membrane (Figure 5C), which completes the transcytosis process. Additional ultrastructural investigations are required, which label both the TfRMAb and the TfR within the endothelial endosomes, so as to determine whether the TfRMAb stays bound to the TfR throughout the transcytotic pathway. At the light microscopic level, the TfRMAb-gold conjugate is only detected within the brain microvasculature (Figure 5A). Since the brain blood volume was cleared by saline, the immunoreactive TfRMAb within the brain capillaries represents TfRMAb within the intra-endothelial volume. No immune reaction is visible in the extravascular brain parenchyma (Figure 5A), which could be interpreted within a model that the TfRMAb only is endocytosed within the endothelium, and without exocytosis into brain parenchyma. However, the rapid transcytosis of either Tf or the TfRMAb across the BBB and into brain parenchyma is demonstrated by autoradiography for Tf (Figure 4B) or for the TfRMAb with the capillary depletion method (76), and these studies show that neither Tf or a TfRMAb remained trapped within the endothelial cytoplasm. The absence of visible immune product in the post-vascular compartment of brain parenchyma following administration of the TfRMAb is not due to a lack of transcytosis through the BBB, but rather is the expected finding of a transcytosis model, which considers the volumetrics of the brain. The TfRMAb visible within the intracellular space of the brain capillary endothelium (Figure 5A) is occupying a volume that is only 0.8 μL/g brain (111). In contrast, the extra-vascular volume of brain is 3 log orders greater than the intra-endothelial volume, or nearly 800 μL/g brain. Therefore, as the TfRMAb exits the intra-endothelial volume and enters into the post-vascular space, the concentration of the TfRMAb undergoes a dilution of ~1,000-fold, which produces a TfRMAb concentration in the post-vascular compartment that is too dilute to detect with light microscopic immune-histochemistry or immune-gold silver staining.

### Methodology for Demonstration of Trans-BBB Passage of a Trojan Horse Candidate

There are multiple methods that have been employed to confirm a given BBB Trojan horse candidate crosses the BBB, and these methods include, (a) immunohistochemical localization of the target receptor on the brain capillary endothelium; (b) ELISA of brain homogenates; (c) *in vitro* BBB models in cell culture; (d) Trojan horse distribution into CSF as a surrogate marker of BBB penetration in brain parenchyma; (e) correction for the brain blood volume; (f) radio-isotopic methods, including light microscopic and film autoradiography; (g) and histochemistry.

#### Immunohistochemistry (IHC)

IHC may be used for two purposes. First, IHC is used to confirm the targeted receptor is expressed at the brain endothelium, which forms the BBB. Second, IHC is used to confirm penetration of the Trojan horse into brain parenchyma following IV administration.

##### IHC localization of targeted receptor at brain endothelium

The IHC in Figure 4A shows expression of the LEPR at the brain capillary endothelium, and is representative of an IHC confirmation of expression of a targeted receptor on the brain capillary endothelium, which forms the BBB. A similar finding is not made for all receptors that are said to be expressed at the BBB. The low density lipoprotein (LDL) receptor (LDLR) is said to act as an RMT system at the BBB on the basis of studies with an vitro BBB model (112). However, the LDLR may be up-regulated in cell culture under conditions of reduced cholesterol availability (113). What is needed is *in vivo* evidence that the LDLR is expressed at the BBB *in vivo*. One indicator for the lack of LDLR function at the BBB *in vivo* is the observation that LDL-bound cholesterol does not enter the brain (114). Immunocytochemistry of mouse brain with an antibody to the insulin receptor shows clear continuous immune staining of the microvascular endothelium, but in the same study, an antibody to the LDLR shows no vascular staining, although the LDLR is expressed on neurons (98). The LDL related protein type 1 (LRP1) is said to function as a BBB RMT system. However, the detection of LRP1 expression in brain shows that immunoreactive LRP1 does not co-localize with an endothelial marker such as PECAM1 (115). Other immunocytochemical studies show localization of LRP1 on the abluminal cells of the brain microvasculature (116), including pericytes (117, 118), and astrocytes (119). Glutathione is said to function as a BBB Trojan horse via binding to the N-methyl-D-aspartate receptor (NMDAR) (120). However, immunocytochemistry of brain shows no expression of the NMDAR at the capillary endothelium, although this receptor is expressed in microvascular pericytes (121). The rabies virus glycoprotein (RVG) peptide is said to function as a BBB Trojan horse via targeting of the nicotinic acetylcholine receptor (nAChR) (122). However, immunocytochemistry of brain *in vivo* shows expression of the nAChR at astrocytes and neurons, but not in brain capillary endothelium (123). In summary, it is difficult to envision how a given Trojan horse could enhance brain delivery if the receptor targeted by that Trojan horse is not expressed on the brain microvascular endothelium *in vivo*, but rather is expressed on brain cells behind the BBB.

##### IHC detection of trojan horse in brain parenchyma

As discussed above with respect to Figure 5A, it is generally not possible to confirm Trojan horse penetration into brain parenchyma with IHC. This is because the Trojan horse undergoes a 1,000-fold dilution as it passes from the intra-endothelial compartment to the extravascular space of brain. The exception is the situation where the Trojan horse is concentrated within the brain parenchyma owing to sequestration of the Trojan horse within a region of interest (ROI) of brain, such as an intra-cranial brain tumor or amyloid plaques in a mouse model of Alzheimer's disease. This sequestration of the Trojan horse within the ROI of brain counteracts the dilution of the Trojan horse that is otherwise widely distributed within the brain.

#### ELISA

It is technically difficult to confirm penetration of a high affinity Trojan horse into brain parenchyma by ELISA measurements of brain extracts obtained following the IV administration of the Trojan horse. This is because the concentration of the Trojan horse in brain is still below the limit of detection (LOD) of most ELISAs. Brain extracts can be concentrated, but this can produce matrix effect artifacts in the ELISA. The injection dose (ID) of a Trojan horse may be increased in an attempt to produce a brain concentration greater than the ELISA LOD. However, the BBB transport of a high affinity Trojan horse is saturated at doses of 4 mg/kg or higher (124), and this saturation of the BBB receptor offsets the higher ID. It may be possible to produce brain concentrations above the LOD of the ELISA following the injection of a very high ID of 50 mg/kg of a low affinity Trojan horse, wherein this high ID of the low affinity Trojan horse does not cause saturation of the BBB receptor.

#### *In vitro* BBB Models

The transport properties of a Trojan horse in an *in vitro* BBB model in cell culture may not translate to the *in vivo* condition, owing to the marked down-regulation of BBB-specific gene expression when brain endothelial cells are grown in cell culture (125). Even the *in vitro* BBB models with high electrical resistance are still 100-fold leaky compared to the BBB *in vivo* (1). Melanotransferrin (p97) was said to be a potential Trojan horse for drug delivery, owing to transport of this protein across an *in vitro* BBB model (126). However, *in vivo* studies showed that p97 does not cross the BBB (127, 128). Angiopep-2, a synthetic 19 amino acid cationic peptide, was said to cross the BBB via LRP1 based on an *in vitro* BBB model (129). However, the brain uptake of a conjugate of angiopep-2 and paclitaxel is low *in vivo*, e.g., 0.29% ID/g (130). Angiopep-2 has failed as a BBB Trojan horse for brain delivery of pegylated liposomes (131) and for delivery of arylsulfatase A, a lysosomal enzyme (132). Cell penetrating peptides (CPP) are highly cationic oligopeptides, which are avidly taken by cells in cell culture, but the brain uptake of these CPPs in the mouse *in vivo* is at the background level (72).

#### CSF

Drug distribution into CSF reflects drug transport across the choroid plexus, which forms the blood-CSF barrier, and should not be used as an index for drug transport across the brain capillary endothelium, which forms the BBB in brain parenchyma (133). The FC5 antibody was isolated from a phage library, and is said to function as a BBB Trojan horse by targeting an orphan receptor (134). Evidence for BBB transport of the FC5 antibody was the observation that this antibody achieves high concentrations in CSF, e.g., 1% of the plasma concentration at 24 h after IV administration (135, 136). However, the concentration of the FC5 antibody within brain tissue, 0.02% ID/g, is very low (136), which indicates the FC5 antibody has minimal penetration of the BBB. These findings indicate the FC5 antibody is a Trojan horse for the blood-CSF barrier at the choroid plexus, but has much lower activity as a Trojan horse for the BBB at the brain capillary endothelium in brain parenchyma.

#### Brain Blood Volume

The brain blood volume is 10–20 μL/g brain in rodents (137). Failure to account for the retention of the drug, or Trojan horse, within this brain blood volume could lead to the erroneous conclusion that the agent crossed the BBB. Aducanumab, the anti-beta amyloid antibody for Alzheimer's disease (AD), was said to cross the BBB because the brain concentration of the antibody increases with higher injection doses. However, the blood concentration of the antibody also increased at the higher IDs (138). The brain/plasma ratio of aducanumab is only ~1 μL/g, which is much less than the cerebral blood volume. Any aducanumab found in the brain may be due solely to incomplete washout of the cerebral blood volume with no antibody penetration of the BBB. In another example of the role of the brain blood volume, the HIRMAb, which does not recognize the rodent insulin receptor, was injected IV in the mouse, and the brain uptake was 0.33% ID/g (60). However, this level of mouse brain uptake of the HIRMAb corresponds to a brain volume of distribution (VD), or brain/plasma ratio, of only 7 μL/g, which approximates the brain blood volume (137). The equivalence of the brain blood volume and the brain VD for the HIRMAb in the mouse indicates this antibody does not cross the BBB in the mouse, because the HIRMAb does not bind to the mouse IR (60).

#### Radio-Isotopic Methods

BBB transport of a Trojan horse can be validated with radio-isotopic methods. Typically, the Trojan horse is radio-iodinated with [^125^I]-iodine and either Iodogen or chloramine T, as either agent will transfer the ^125^I radionuclide to a tyrosine residue on the Trojan horse via an oxidative chemical reaction. Following the IV administration of the labeled Trojan horse, brain radioactivity is measured, and the brain volume of distribution (VD), or brain/plasma ratio, is determined over time points up to 60 min. Alternatively, the brain uptake can be expressed as %ID/g brain. There are potential artifacts with this approach, which are exemplified in the case of the brain uptake of epidermal growth factor (EGF). Following oxidative iodination of EGF, and IV administration of the [^125^I]-EGF, the plasma radioactivity that is precipitable with trichloroacetic acid (TCA) falls rapidly over the course of 60 min (139). The EGF is rapidly taken up by peripheral tissues, followed by intracellular degradation with release of [^125^I]-tyrosine, and the [^125^I]-tyrosine is exported to plasma, which accounts for the increase in plasma radioactivity that is TCA-soluble. These metabolites may then enter brain, which could be interpreted as evidence for the brain uptake of the [^125^I]-EGF (139). In this setting, the measurement of brain radioactivity as an index of BBB transport of EGF is an artifact, which is demonstrated by the use of an alternative radio-labeling of EGF with the ^111^In radionuclide. If the EGF is conjugated with diethylenetriaminepentaacetic acid (DTPA), which attaches to EGF lysine residues, the EGF may be radiolabeled owing to DTPA chelation of the [^111^In] radionuclide. The rat brain uptake of [^111^In]-EGF is 10-fold lower than the brain uptake of [^125^I]-EGF (139). When the [^111^In]-EGF is degraded in peripheral tissues, the lysyl-DTPA-[^111^In] is not released back to blood, but rather is sequestered within peripheral tissues (140). This was confirmed with [^111^In]-EGF as HPLC of plasma showed no low MW radio-labeled metabolites in the rat (141). These studies indicate the superiority of the [^111^In]-radiolabeling approach over the oxidative iodination with [^125^I]-iodine. The [^111^In] radionuclide is often not readily available, and there is an alternative to stable radiolabeling that is more widely available, and this is the [^125^I]-Bolton-Hunter reagent, which attaches the radionuclide via a non-oxidative process to lysine residues on the Trojan horse. Unlike [^125^I]-tyrosine, which does cross the BBB, the [^125^I]-Bolton-Hunter-lysine does not cross the BBB (142). Therefore, brain radioactivity measurements reflect brain uptake of the Trojan horse, and not metabolites, when the Trojan horse is radiolabeled with the [^125^I]-Bolton-Hunter reagent.

##### Film autoradiography

**Figure 6:**
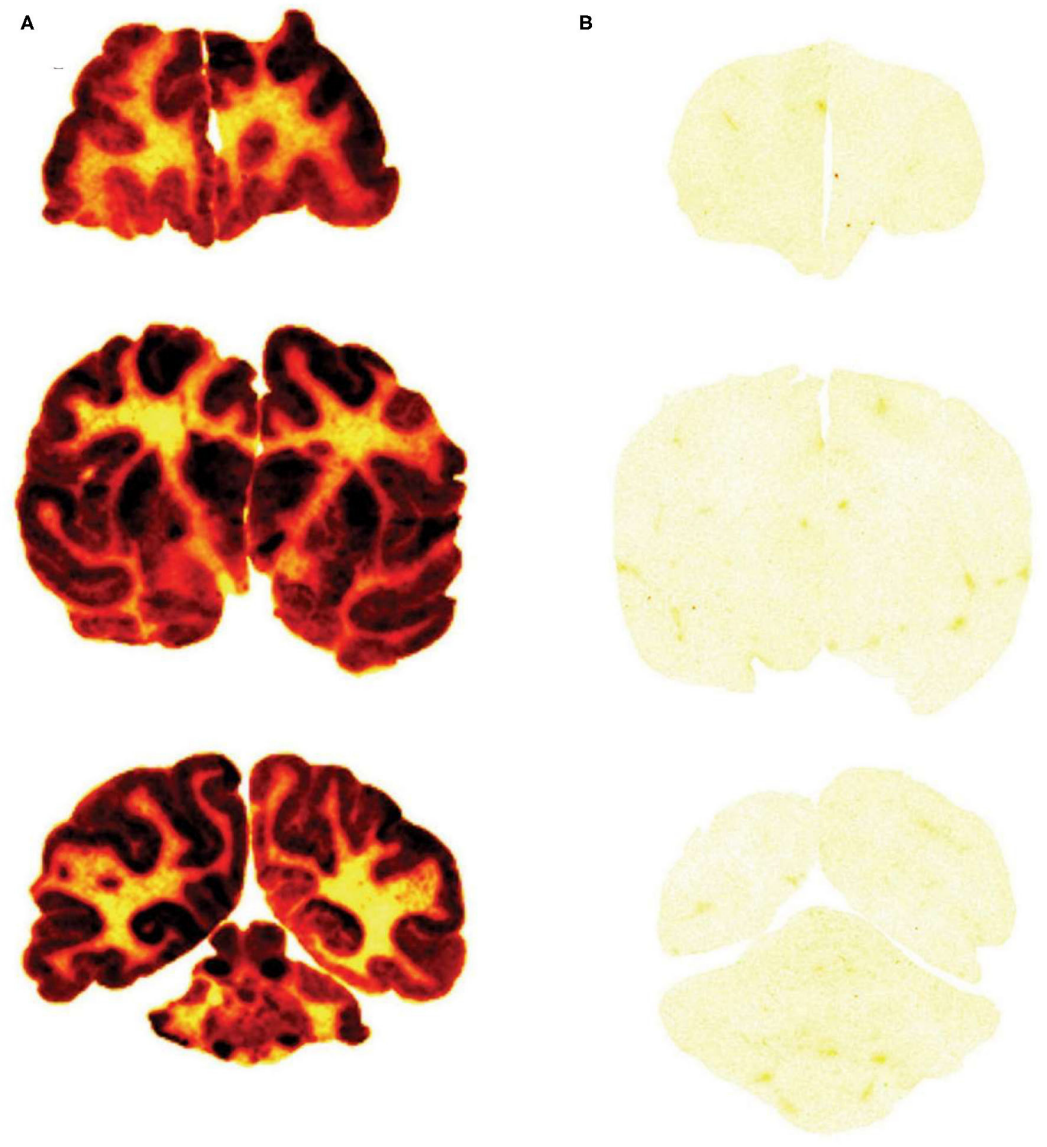
Film autoradiogram of 20 μm coronal sections of rhesus monkey brain removed 120 min after IV injection of the HIRMAb-IDS fusion protein **(A)** or IDS enzyme **(B)**. The forebrain section is on the top, the midbrain section is in the middle, and the hindbrain section with cerebellum is on the bottom. Scans produced after labeling of the HIRMAb-IDS fusion protein or IDS with the [^125^I]-Bolton-Hunter reagent. Xrays exposed for 7 days. HIRMAb, human insulin receptor monoclonal antibody; IDS, iduronate 2-sulfatase. Fusion of the IDS enzyme, which alone does not cross the BBB **(B)**, to the HIRMAb enables global penetration of the lysosomal enzyme throughout the primate brain **(A)**. Reproduced with permission from Boado et al. (142).

The brain uptake of a Trojan horse candidate labeled with the [^125^I]-Bolton-Hunter reagent can also be assessed with film autoradiography following the frozen sectioning of brain removed after the IV administration of the Trojan horse. The method is exemplified by a study in the Rhesus monkey (142). Iduronate 2-sulfatase (IDS), the lysosomal enzyme mutated in Hunter syndrome, was labeled with the [^125^I]-Bolton-Hunter reagent and the labeled IDS was injected IV in the primate. In parallel, a fusion protein of IDS and a MAb against the human insulin receptor (HIR) was labeled with the [^125^I]-Bolton-Hunter reagent, and the labeled HIRMAb-IDS fusion protein was injected IV in a second Rhesus monkey. The brains from the monkeys were removed 2 h after IV injection, and 20 micron frozen sections of brain were prepared and applied to x-ray film (142). The biodistribution in brain of the HIRMAb-IDS fusion protein is shown Figure 6A, and the brain uptake of the IDS enzyme, which does not cross the BBB, is shown in Figure 6B. This film autoradiography shows global penetration of the monkey brain of the HIRMAb-IDS fusion protein, owing to RMT of the fusion protein on the primate BBB insulin receptor. The plasma concentration of either the IDS or the HIRMAb-IDS fusion protein is very low at 2 h. Therefore, the only compartments that could retain the HIRMAb-IDS fusion protein is either the intra-endothelial compartment or the post-vascular brain compartment. If the fusion protein was only retained in the intra-endothelial compartment, the robust brain scan shown in Figure 6A could not be observed. This is because the intra-endothelial compartment of brain is only 0.1% of the brain volume, as discussed above. Moreover, transcytosis of the HIRMAb enzyme fusion protein was confirmed with the capillary depletion method (142). A more definitive test of Trojan horse penetration into brain parenchyma beyond the BBB is light microscopic emulsion autoradiography of brain following IV administration of the [^125^I]-Bolton-Hunter reagent-labeled Trojan horse fusion protein, as demonstrated for a fusion protein of the HIRMAb and the lysosomal enzyme, arylsulfatase A (ASA) (143). The emulsion autoradiography study showed rapid delivery of the HIRMAb-ASA fusion protein through the BBB followed by widespread distribution into brain parenchyma (143), similar to that shown for transferrin (Figure 4B).

##### Positron emission tomography

Brain uptake of proteins radiolabeled with positron emitting isotopes may be visualized *in vivo* with positron emission tomography (PET). Plasma proteins were conjugated with a chelator moiety, 1,4,7,10-tetraazacyclododecane-1,4,7,10-tetraacetic acid (DOTA), which enabled radiolabeling with the PET isotope, ^64^Cu (144). Brain uptake of plasma proteins was visualized in mice with a microPET scanner, and these studies provided initial evidence that BBB transcytosis is reduced with aging in 22 month old mice (144).

#### Histochemistry

BBB transport of THLs carrying a LacZ β-galactosidase expression plasmid DNA was confirmed in the rat with the OX26 MAb against the rat TfR (49), in the mouse, with the 8D3 MAb against the mouse TfR (48), and in the primate with the 83–14 MAb against the HIR (50), which cross reacts with the Old World primate insulin receptor (51). An example of X-gal histochemistry of brain removed 2 days after the IV administration of the TfRMAb-targeted THL encapsulating the LacZ expression plasmid DNA is shown in Figure 3 for the mouse (47). This global expression of the LacZ gene in brain shows that the MAb on the surface of the THL triggers RMT across the BBB, followed by receptor-mediated endocytosis into brain cells, followed by triage to the nuclear compartment for gene expression. The replacement of the receptor-specific Trojan horse MAb with a non-specific MAb results in no expression of the LacZ gene following THL administration in the mouse (48) or the rat (49), which shows gene expression in brain is solely a function of the receptor specificity of the Trojan horse on the surface of the THL.

BBB Trojan horses that have been developed are of 2 different types of molecules: peptides that bind a specific BBB receptor, and MAb's that bind exofacial epitopes on a specific BBB receptor, and this binding enables RMT of the Trojan horse across the BBB.

### Peptide Trojan Horses

#### Insulin Receptor Peptides

Insulin is a 5 kDa peptide comprised of two chains (A chain and B chain), which are covalently linked via disulfide bonds. The use of insulin as a Trojan horse domain in a genetically engineered fusion protein first requires the two-chain mature insulin be converted into a biologically active single chain form of insulin. Use of the single chain proinsulin precursor is not possible owing to the very low affinity of proinsulin for the insulin receptor. This problem was remedied by insertion of a dodecapeptide between the A and B chains of insulin, creating a biologically active single chain form of insulin. This single chain insulin was then fused to albumin to create an insulin-albumin fusion protein, which retained high affinity for binding to the HIR, as the ED50 of binding of insulin vs. the insulin-albumin fusion protein was 1.1 and 7.4 nM, respectively (145). The use of the single chain insulin could be extended to the engineering of insulin-Fc fusion proteins for BBB delivery using the single chain form of insulin. However, the disadvantage of using insulin as a Trojan horse is that hypoglycemia could be a limiting side effect.

#### Transferrin Receptor Peptides

Transferrin (Tf) has been used as a BBB Trojan horse for BBB delivery of a pegylated dendrimer (146), for delivery of pegylated liposomes (119), as well as for delivery of plasmid DNA conjugated to cationic lipoplexes (147). However, the problem with using Tf as a Trojan horse is that this *exogenous* Tf Trojan horse must compete with the *endogenous* Tf in plasma for binding to the TfR on the BBB. Tf exists in plasma in very high concentration of 2–4 mg/mL, which is equivalent to 25–50 μM (148). This level of endogenous Tf in the circulation is >2,000-fold higher than the KD of Tf binding to the BBB TfR (99). Therefore, the BBB TfR is >99% saturated by endogenous Tf, which greatly restricts access of the exogenous Tf to the BBB TfR. Even in cell culture, which includes 10% serum, the concentration of medium endogenous Tf is >1,000 nM, which will inhibit the uptake of exogenous Tf by endothelial cells tested with *in vitro* BBB models, owing to >99% saturation of the endothelial TfR by the Tf in 10% serum. For these reasons, Tf should not be used as a control Trojan horse for *in vitro* BBB models performed in the presence of even small amounts of serum.

#### IGF Receptor Peptides

Both IGF-1 and IGF-2 undergo saturable transport across the BBB, as demonstrated by carotid arterial infusion of the peptides in the absence of serum (96), and this transport is mediated by the BBB IGFR receptor (101). However, both IGF peptides are >99% bound by IGF binding proteins (IGFBP) (149). The binding of the IGFBPs to the IGFs blocks the BBB transport *in vivo*. This was illustrated with the engineering of a fusion protein of IGF2 and the lysosomal enzyme, NAGLU. The IGF2-NAGLU fusion protein was biologically active following the intra-cerebral injection of the fusion protein, but did not penetrate the brain following IV administration (150). The IGF2-NAGLU fusion protein in blood did not penetrate the BBB, presumably due to the inhibitory effect of IGFBP binding of the growth factor.

#### LRP1 Receptor Peptides

Angiopep-2 was proposed as a BBB Trojan horse owing to transport across an *in vitro* BBB model, and LRP1 was said to be the target receptor (129). Setting aside the lack of expression of LRP1 on the capillary endothelium, as discussed above, angiopep-2, a 19-amino acid peptide, has very low affinity of binding to LRP1, as demonstrated with a binding assay using recombinant LRP1 extracellular domain (ECD). The LRP1 has 4 extracellular domains, I-IV, and most LRP1 ligands bind domains II and IV (151). However, the binding ED50 of angiopep-2 to domains II and IV was ≥1,000 nM (151). This binding avidity is nearly 3 log orders of magnitude lower than the binding of insulin, the IGFs, leptin, or Tf to the respective BBB receptor (1). In another study, no measurable binding of angiopep-2 to either domain II or IV of LRP1 could be detected (152).

Certain cationic oligopeptides derived from the sequence of human apolipoprotein E (apoE) are low affinity ligands for LRP1 (153). ApoE (130–149) is a 20 amino acid cationic peptide, which corresponds to amino acids (130–149) of human apoE (AAB59397), where the amino acid numbering starts at the amino terminal Lys, and does not include the 18 amino acid signal peptide. ApoE (130–149) bound to LRP1 domains II and IV with ED50 values of 51 and 129 nM. ApoE (141–155) was synthesized as a 30 amino acid peptide with a repeat sequence of amino acids (141–155) of human apoE, and this cationic peptide bound LRP1 domains II and IV with ED50 values of 118 and 190 nM, respectively (153). This low affinity binding is expected for oligopeptide fragments of a much larger protein, e.g., apoE. The apoE (130–149) peptide was co-administered with a lysosomal enzyme, tripeptidyl peptidase I (TPP1), to enhance BBB delivery of the enzyme via electrostatic interactions between the anionic enzyme and the cationic apoE peptide (79). However, it was necessary to administer intravenously the apoE peptide at high doses of 10 mg/kg, which proved to be toxic resulting in injection-related reactions and death (79). Such toxicity is expected for the administration of cationic proteins, as described above in the absorptive-mediated transcytosis section.

ApoE peptides, designated ApoE-1 and ApoE-2 were fused to the lysosomal enzyme, arylsulfatase A (ASA), in an attempt to deliver this lysosomal enzyme across the BBB (132). The ApoE-1 peptide is a cationic peptide, which corresponds to amino acids 130–152 of human apoE, which overlaps with the ApoE (130–149) peptide of Croy et al. (153). The ApoE-2 peptide is an 18 amino acid cationic peptide comprised of a repeat sequence of amino acid (141–149) of human apoE, which overlaps with the ApoE 141–155 peptide of Croy et al. (153). In addition, ASA was fused to tat, angiopep-2, or a 38 amino acid cationic peptide corresponding to amino acids 264–301 of human apolipoprotein B100 (ApoB: AAA59750). The fusion of angiopep-2, tat, the apoB peptide, or the apoE-1 peptide to ASA had no effect on delivery of the enzyme to brain. Fusion of the apoE-2 peptide caused a modest increase in brain enzyme activity at a high IV injection dose of 20 mg/kg (132). Given the strong cationic charge of the apoE-2 peptide, the mechanism of transport across the BBB of the apoE-2/ASA fusion protein may have been a charge related absorptive mediated endocytosis process, rather than receptor-mediated transport. The apoE (130–149) peptide of Croy et al. (153), and the apoE-2 peptide of Bockenhoff et al. (132) overlap with the COG-133 peptide, which is a 17 amino acid cationic peptide that corresponds to amino acids 133-149 of human apoE, and which was used by van Rooy et al. (131) to enhance the BBB transport of pegylated liposomes across the BBB. However, the COG-133 peptide, angiopep-2, and the CRM197 protein had no effect on the BBB delivery of the pegylated liposome. Only the RI7-217 TfRMAb enhanced brain delivery of the liposomes (131).

Lactoferrin (Lf) is a major iron binding protein in milk. Lf is said to cross the BBB based on an *in vitro* BBB model (154), and Lf has been used as a BBB Trojan horse for brain delivery of pegylated liposomes (155, 156). Lf was shown to be a ligand for LRP1 (157), which is not expressed on brain endothelium *in vivo* as discussed above. More recent work has identified the Lf receptor (LfR) as intelectin-1 (ITLN1, NP_060095) (158), a 313 amino acid glycoprotein with a 16 amino acid signal peptide, also known as omentin. Intelectin-1 is expressed in the intestinal epithelium, and may mediate uptake of milk-derived Lf. However, there are no studies demonstrating expression of intelectin-1 at the BBB. In ischemia, parenchymal intelectin-1 promotes neovascularization (159). Irrespective of the actual receptor that mediates brain uptake of Lf, the brain uptake of Lf after IV administration is very low, and is only 0.015%ID/g brain in the rat (160).

#### NMDA Receptor Peptides

Glutathione (GSH) is a tripeptide, pyroglutamyl-cysteinyl-glycine, and is said to act as a BBB Trojan horse for the delivery of pegylated liposomes (161, 162). GSH is proposed to be a ligand for the BBB NMDA receptor (NMDAR) (120). However, the NMDAR is not localized to the brain endothelium by immunohistochemistry, but rather is expressed in pericytes (121). A peptide Trojan horse that targets a pericyte receptor cannot act as a BBB Trojan horse, because there is no mechanism for the peptide to first cross the endothelial barrier. Alternatively, GSH transport across the BBB may be carrier-mediated via ABC types of active efflux systems, such as multidrug resistant protein 1 (MRP1, ABCC1) (163). However, MRP1 is not detected in brain capillaries (164), and immunoreactive MRP1 co-localizes with GFAP on astrocyte endfeet (165). Any GSH in the blood may be rapidly degraded at the brain endothelial surface by γ-glutamyl transpeptidase (GGTP), which is an ectoenzyme on the endothelial luminal membrane (166).

#### Nicotinic ACh Receptor Peptides

A 29 amino acid peptide derived from the rabies virus glycoprotein (RVG) is said to act as a BBB Trojan horse by targeting the nicotinic acetylcholine receptor (nAChR) on the BBB (122). However, immunocytochemistry of brain shows the nAChR is expressed in astrocytes and neurons, but not in endothelium in brain (123). The 29 amino acid RVG peptide was fused via a three amino acid linker to a sequence of nine arginine residues (122). Therefore, this 41 amino acid peptide was highly cationic, and any BBB transfer may be due to an absorptive-mediated transport process observed for polycations, as discussed above.

#### HBEGF Peptides

CRM197, a non-toxic mutant of the dipheria toxin (DT) originally developed as an immune adjuvant (167), is said to act as a BBB Trojan horse for the delivery of pegylated liposomes (168), or nanoparticles (169). The DT receptor is the membrane bound form of the heparin binding EGF-like growth factor (HB-EGF) precursor protein. Immunocytochemistry with an antibody against the HB-EGF precursor shows abundant expression in neurons and oligodendrocyte precursors, but minimal, if any endothelial expression of HB-EGF in brain (170). The DT binds HB-EGF precursor protein with high affinity in humans and primates, but with very low affinity in rodents (171). CRM197 does not bind the HB-EGF in the mouse (172). However, the DT binds the HB-EGF in guinea pigs with high affinity (173). CRM197 toxicity was tested in guinea pigs, and the IV administration of 50–500 μg/kg CRM197 caused BBB disruption, and ultrastructural changes in the brain endothelium (174).

#### Orphan Receptor Phage Peptides

A fd phage library of 15 amino acid random sequences was screened for brain penetrating peptides that could be used as a Trojan horse (175). This study led to the identification of a 15-mer GLA peptide. The GLA peptide was synthesized with a cysteine residue to enable conjugation to pegylated liposomes with a maleimide group (176). However, these GLA-pegylated liposomes were taken up poorly by brain human hCMEC/D3 cells in culture. It was hypothesized that the GLA peptide, as a stand-alone 15-mer, exists in an altered conformation as compared to the 15-mer sequence that is part of the much larger p3 minor coat protein of the fd filamentous phage. Therefore, the GLA peptide as part of the 240 amino acid amino-terminal domain of the p3 protein was synthesized and this large peptide was designated the p3-GLA peptide (176). Pegylated liposomes produced with the larger p3-GLA peptide were taken up by hCMEC/D3 cells to a greater extent than liposomes produced with the 15-mer GLA peptide. The receptor on the BBB being targeted by the GLA peptide has not been identified.

### Monoclonal Antibody Trojan Horses

A BBB CMT system is a transmembrane pore that does not leave the plasma membrane during the transport cycle. Some CMT systems are exceptions, as the insulin-dependent GLUT4 glucose transporter is sequestered in the intracellular space in the absence of insulin (177). In contrast, a RMT system involves ligand binding to the receptor, which is then followed by endocytosis of the receptor/ligand complex into the intracellular endosomal compartment. Consequently, a MAb that binds an exofacial epitope on the receptor, which is spatially removed from the endogenous ligand binding site, may “piggy-back” on the receptor endocytosis and enter the cell along with the receptor/ligand complex, as originally demonstrated for the LDL receptor (178). This MAb may then separate from the endosomal system and undergo exocytosis into the brain interstitial space, as illustrated for a TfRMAb in Figure 5C. The MAb should be an ‘endocytosing antibody,’ which is an antibody that binds an epitope on the receptor and does not interfere with the endocytosis process. Not all receptor-specific antibodies are endocytosing antibodies. For example, trastuzumab, an antibody against the HER2 receptor, is not internalized by the target cell following receptor binding, and is localized to the cell membrane (179).

#### Insulin Receptor Monoclonal Antibodies

The 83–14 antibody is a mouse MAb against the human insulin receptor (HIR), and this antibody cross-reacts with the BBB insulin receptor of Old World primates such as the Rhesus monkey (51), but does not react with the BBB insulin receptor in mice (60). This antibody, designated the HIRMAb, binds with high affinity to the HIR on isolated human brain capillaries, followed by endocytosis into the endothelium (51). The HIRMAb has been genetically engineered and both chimeric (180) and humanized forms of the antibody have been produced (181). The amino acid sequences of the variable region of the heavy chain (VH) and the variable region of the light chain (VL) have been published (181), which enables the custom production of a recombinant form of this antibody by commercial vendors (53). Fusion proteins of the chimeric HIRMAb have been engineered for neurotrophins, decoy receptors, lysosomal enzymes, and therapeutic antibodies, and in most cases the bi-functionality of the fusion protein is retained (182). A fusion protein of the chimeric HIRMAb and human IDUA has been genetically engineered, and the plasma pharmacokinetics of this HIRMAb-IDUA fusion protein, designated valanafusp alpha, have been reported following the IV infusion of 1–6 mg/kg doses of the fusion protein in humans (183). Valanafusp alpha is the first BBB Trojan horse to enter human clinical trials, and the safety and efficacy of 12 months of treatment of children with Mucopolysaccharidosis (MPS) Type I (MPSI) with 1–6 mg/kg of valanafusp alpha have been described (184). The incidence of hypoglycemia or infusion related reactions was <2% over the course of 500 IV infusions. The decline in developmental quotient that occurs with MPSI, which is a measure of the intellectual disability, was halted with chronic valanafusp alpha treatment (184). In addition to the engineering of BBB penetrating IgG fusion protein drugs for human neural disease, the HIRMAb has also be used as the Trojan horse for delivery of THLs to the primate brain *in vivo* (50). Manufacturing of HIRMAb-targeted THLs, including the lyophilization of these formulations for long term storage, is discussed in the Manufacturing section below.

#### Transferrin Receptor Monoclonal Antibodies

The mouse OX26 MAb against the rat TfR was shown to bind to the microvascular endothelium of brain by immunohistochemistry (185), and the expression of the TfR at the human BBB was demonstrated by receptor binding assays with microvessels isolated from human autopsy brain (99). The OX26 TfRMAb enters rat brain rapidly following IV injection (186). The amino acid sequences of the VH and VL of the OX26 antibody have been published (187), which enables the custom production of recombinant versions of this antibody by commercial vendors. The OX26 antibody does not cross react with the TfR in the mouse (124), but the rat 8D3 or rat RI7-217 antibodies against the mouse TfR rapidly penetrate the mouse brain following IV administration (124). The amino acid sequences of the VH and VL of the 8D3 antibody have been published (188), which enables the custom production of recombinant forms of this antibody by commercial vendors, as reported recently (54). The mouse 128.1 MAb against the human TfR has been genetically engineered as a humanized TfRMAb, and shown to rapidly enter both brain and CSF of the Rhesus monkey following IV administration (189). The TfRMAb enters brain via RMT across the BBB within brain parenchyma. In parallel, the TfRMAb enters CSF via RMT across the choroid plexus, or blood-CSF barrier. The rapid transport of the TfRMAb across the choroid plexus produces a CSF/plasma ratio of 4.8% at 23 h after IV administration (189). The rapid entry of the TfRMAb into CSF is due to the very high expression of the TfR1 at the choroid plexus (190).

Genetically engineered fusion proteins of the chimeric 8D3 TfRMAb and different classes of biologics, including neurotrophins, lysosomal enzymes, decoy receptors, and therapeutic antibodies have been described, as well as the treatment of mouse models of neural disease with these TfRMAb fusion proteins (182). MPSI mice were treated chronically with the TfRMAb-IDUA fusion protein, and this produced a 70% reduction in brain lysosomal inclusion bodies (191). MPSIIIA mice were treated chronically with a fusion protein of the TfRMAb and the enzyme mutated in MPSIIIA, N-sulfoglucosamine sulfohydrolase (SGSH), and this treatment produced a 70% decline in brain heparan sulfate (HS) as determined by liquid chromatography mass spectrometry (192).

A new treatment of the brain in MPSII was engineered by Sonoda et al. (193). Human iduronate 2-sulfatase (IDS), the enzyme mutated in MPSII, was fused to a humanized MAb against the human TfR, which has high affinity for the human TfR with a KD of 0.12 nM (193). This antibody did not cross react with the mouse TfR. Therefore, a double transgenic mouse was engineered with an IDS knock-out combined with a knock-in of the human TFRC (TfR1) receptor. With this novel mouse model, the TfRMAb-IDS fusion protein engineered for treatment of humans with MPSII could be validated in a mouse model of MPSII. This high affinity TfRMAb-IDS fusion protein has entered into human clinical trials, and has an excellent safety profile at infusion doses of 1–2 mg/kg. Treatment causes a 31% decrease in CSF HS in human subjects with MPSII (194). Reduction in CSG glycosaminoglycans, such as HS, is expected given the rapid uptake of TfRMAb's into the CSF (189). In contrast, chronic treatment of subjects with MPSI with the HIRMAb-IDUA fusion protein did not reduce HS in CSF (184). The differential response of the CSF marker to treatment with a HIRMAb vs. a TfRMAb is attributed to the 16-fold higher expression of the TfR, as compared to the insulin receptor, at the choroid plexus (190). In contrast, the expression of the TfR and the HIR at the human BBB is comparable (195). Owing to the differential expression of the TfR and the IR at the BBB vs. the blood-CSF barrier, the TfRMAb penetrates the CSF to a much greater extent than the HIRMAb. However, both the TfRMAb and the HIRMAb penetrate the BBB and enter into brain parenchyma of the Rhesus monkey at comparable rates (180, 189).

#### Low Affinity Transferrin Receptor Monoclonal Antibodies

The failure to observe immune reaction product in brain parenchyma following the IV administration of a TfRMAb led to the hypothesis that the high affinity of the TfRMAb for the BBB TfR caused sequestration of the TfRMAb within the capillary endothelium, with minimal transcytosis into brain (196). This model does not explain why Tf and a TfRMAb undergo BBB transcytosis *in vivo* at comparable rates (76). The comparable transcytosis of Tf and a TfRMAb is expected since both Tf and the TfRMAb have the same high affinity of binding to the TfR. The KD of binding of Tf to the TfR is 5 nM (99), whereas the KD of binding of a TfRMAb such as the 8D3 MAb against the mouse TfR is 2.3 nM (188). Based on the model that the high affinity TfRMAb does not transcytose through the BBB, genetic engineering was used to progressively lower the affinity of the TfRMAb for the TfR, which resulted in a panel of TfRMAb's where the ED50 of TfR binding was raised from 1.7 nM to 6.9 nM to 65 nM to 111 nM, and these antibodies were designated anti-TfR^A^, anti-TfR^B^, anti-TfR^C^, and anti-TfR^D^, respectively (196). The brain uptake of these TfRMAbs at low injection doses (ID) was inversely related to the ED50 of antibody binding to the TfR; at low doses, the high affinity TfRMAb had a higher brain uptake than the low affinity TfRMAb. However, when the ID was raised to 20 mg/kg and the brain uptake was measured at 24 h after IV administration, the brain uptake was higher with the low affinity anti-TfR. This observation suggested low affinity TfRMAbs were preferred BBB Trojan horses, as compared to a high affinity TfRMAb (196). However, these findings at high injection doses are the expected results, and do not provide evidence for the lack of transcytosis of a high affinity TfRMAb through the BBB. The high ID of 20 mg/kg selectively saturates the TfR binding site on the BBB for the high affinity anti-TfR, but not for the low affinity anti-TfR. The use of a low affinity anti-TfR as a BBB Trojan horse forces the drug developer to administer higher injection doses to offset the reduced affinity of the TfRMAb for the TfR. These high IDs lower the therapeutic index of the anti-TfR. The administration of certain therapeutic compounds at an ID of 20 mg/kg will cause toxicity, as reviewed previously (197). Subsequently, the hypothesis that the low affinity TfRMAb was the preferred Trojan horse was modified, and high affinity TfRMAbs were proposed as preferred delivery systems (198).

##### Dissociation rate of low affinity antibodies

The affinity of an antibody for a BBB receptor is inversely related to the molar dissociation constant (KD), where KD = k_off_/k_on_, and k_off_ (sec^−1^) is the dissociation rate constant, and k_on_ (M^−1^sec^−1^) is the association rate constant. The KD is determined by the k_off_, as the k_on_ is relatively unchanged despite wide alterations in the KD (199). The KD, and k_off_, of a monoclonal antibody were varied >600-fold by point mutations in the complementarity determining regions (CDR) of either the VH or the VL of the antibody. The high affinity form of the antibody had a KD of 8 nM and k_off_ of 7 × 10^−4^ sec^−1^. Mutant forms of the antibody had a KD of 20, 163, 357, and 643 nM, and these mutants had k_off_ values of 18 × 10^−4^ s^−1^, 186 × 10^−4^ s^−1^, 294 × 10^−4^ s^−1^, and 622 × 10^−4^ sec^−1^, respectively, which corresponds to dissociation half-times of 1,000 s for the high affinity antibody and 390, 37, 23, and 11 s for the low affinity mutants, respectively. Endocytosis of the TfR-bound antibody requires that the rate of dissociation from the receptor is sufficiently slow so that the antibody is still bound to the receptor during the endocytosis process. The dissociation half-times of low affinity antibodies, e.g., antibodies with a KD > 100 nM, are 11–37 s, and such loosely bound antibodies may dissociate from the receptor and be swept back into the circulation before endocytosis of the antibody/receptor complex takes place. The half-time of internalization of the TfR is 3.5 min in cultured HepG2 cells (200) and is 6.1 min in cultured CHO cells (201). Although the rate of endocytosis of the TfR/TfRMAb complex at the BBB *in vivo* is not known, it is likely the rate of this process is slower than the rate of dissociation from the TfR of a low affinity TfRMAb.

#### Basigin Monoclonal Antibodies

A panel of MAbs against basigin, also known as CD147, have been prepared as alternative antibody-based BBB Trojan horses (202). Basigin was originally identified at the brain microvasculature as neurothelin or the HT7 protein, an immunoglobulin-like membrane glycoprotein (203). Basigin/CD147 participates in the carrier-mediated transport (CMT) of monocarboxylic acids (MCA), such as lactate, pyruvate or ketone bodies, as CD147 forms a hetero-dimer with the type 1 MCA transporter (MCT1) (204), and CD147 facilitates MCT1 insertion in the plasma membrane (205). CD147 is present in high amounts in microvessels isolated from mouse brain (206), but is undetectable in human brain microvessels (195). Given that CMT systems generally do not undergo endocytosis, it is not clear if basigin MAbs will be effective BBB delivery agents. Basigin/CD147 is the receptor for the malaria parasite, *plasmodium falciparum*, and basigin mediates the parasite invasion into tissues (207). However, the *P. falciparum* does not invade the brain parenchyma or cross the intact BBB; instead human cerebral malaria is caused BBB disruption and cerebral hemorrhage (208).

#### Phage Library Monoclonal Antibodies

A non-immune human single chain Fv (ScFv) Fd phage library (209) was bound to primary cultures of rat brain endothelial cells following initial subtraction with endothelial lines from heart and lung. This work led to the identification of ScFv15 and ScFv38 which localized to rat brain capillary endothelium based on confocal microscopy (210). The work did not describe the putative binding site on the BBB, nor the affinity constant of binding to the putative receptor. Since there is marked down-regulation of BBB gene expression in cell culture (125), the use of culture endothelium to clone novel BBB-binding antibodies is less sensitive than screening with the *in vivo* brain uptake of the phage ScFvs. The latter approach was described by Stutz et al. (211), and this work led to the isolation of ScFv40 based on capillary binding in rat brain using confocal microscopy. However, such work needs to be extended with the identification of the BBB binding site, using methods such as affinity cross-linking of the ^125^I-labeled antibody and freshly isolated brain microvessel membranes. Other studies needed to assess the potency of the ScFv include the measurement of the uptake of the labeled ScFv by rat brain following IV administration.

An example of screening a hyper-immune human ScFv library was reported by Zabeau et al. (212). Llamas were immunized with the mouse LEPR extracellular domain (ECD). Blood lymphocyte RNA was used to produce cDNA. Nanobody sequences were amplified and cloned into a phage display library, and antibodies were selected by panning with the mouse LEPR ECD. The goal was to isolate neutralizing anti-LEPR antibodies as therapeutics that enhance weight gain via the LEPR/leptin interaction. Such neutralizing antibodies would not be suitable antibodies for drug delivery across the BBB via transport on the BBB LEPR, because the antibody would interfere with the transport of endogenous leptin across the BBB. However, non-neutralizing antibodies could have been isolated by panning the library with a complex of the mouse LEPR ECD and mouse leptin. The receptor bound leptin ligand would cover the leptin binding site and eliminate the isolation of neutralizing antibodies that bind the leptin binding site on the LEPR. The screening of ScFv phage libraries with complexes of the IR ECD/insulin, the TfR1 ECD/Tf, the LEPR ECD/LEPR, or the IGF1R ECD/IGF1 could be performed, so as to produce panels of novel antibodies, which may prove to have useful properties as a BBB Trojan horse.

### TfRMAb and Effector Function

There is an interaction between the TfR and the Fc gamma receptor (FcγR) following the administration of a TfRMAb, owing to binding of the Fc region of the antibody to the FcγR. The acute administration of a TfRMAb caused severe first injection reactions (FIR) and suppressed reticulocytes (213). These FIRs were not observed in monkeys with a TfRMAb engineered with the N297G mutation in the Fc region, which eliminates glycosylation of the IgG (198). Removal of the Fc carbohydrate eliminates interaction of the IgG with the FcγR, but has no effect on IgG binding to the neonatal Fc receptor (FcRn) (214, 215). These effector function toxicities were not observed for the HIRMAb in monkeys (216) or humans (184), and are peculiar to TfRMAb interactions with the FcγR. The N297G mutation was said not to alter the plasma pharmacokinetics of the aglycosylated TfRMAb following the IV administration in the mouse of a very high injection dose, 50 mg/kg (217). Such high Trojan horse injection doses (ID) are required for low affinity Trojan horses (196). However, the ID for a high affinity TfRMAb is much lower, e.g., 1–3 mg/kg, and plasma pharmacokinetics of a high affinity aglycosylated TfRMAb should be performed at the expected therapeutic injection dose. This was done for a high affinity aglycosylated TfRMAb erythropoietin (EPO) fusion protein (218). Removal of the Fc glycosylation site from a high affinity TfRMAb had a profound effect on the plasma pharmacokinetics of the TfRMAb-EPO fusion protein in the mouse, particularly after the subcutaneous (SQ) route of administration that would be used for chronic treatment of neurodegeneration. Following the SQ administration of 3 mg/kg of the native TfRMAb-EPO fusion protein, or the same fusion protein with the N292G mutation, designated TfRMAb-N292G-EPO, there was a 93-fold increase in the plasma clearance of the N292G mutated fusion protein as compared to the native TfRMAb-EPO fusion protein (218). The maximal plasma concentration, Cmax, of the mutant fusion protein was reduced 114-fold compared to the native TfRMAb-EPO fusion protein. The much lower plasma Cmax of the TfRMAb-N292G mutant Trojan horse is due to the very fast plasma clearance of the aglycosylated TfRMAb-EPO fusion protein. Therefore, the use of aglycosylated TfRMAbs may have reduced effector function, but also exhibit an unacceptable plasma pharmacokinetic profile, particularly after SQ administration.

#### Antibody Fucose Content

First injection reactions (FIRs) have also been reported following the IV injection of pegylated immunoliposomes conjugated with the RI7-217 MAb (67), which is a rat MAb against the mouse TfR that crosses the mouse BBB (124). FIRs are attributed to effector function of the constant domain of the IgG, although FIRs could also be caused by endotoxin contamination in the antibody preparation. Endotoxin contamination can be tested with the Limulus amoebocyte lysate (LAL) colorimetric assay. The endotoxin content of the antibody should be <1 endotoxin units (EU)/mg protein. Effector function mediated FIRs are caused by binding of the oligosaccharide moiety of the MAb to the FcγR. This oligosaccharide moiety is linked to a single asparagine residue in the Fc region of the heavy chain, and is comprised of multiple monosaccharides, including fucose, galactose, mannose, sialic acid, and N-acetylglucosamine (219). The most critical monosaccharide mediating the IgG binding to the FcγR is fucose (220). IgG with high fucose content has low FcγR binding and low effector function, whereas IgG with low fucose has high FcγR binding and high effector function (221, 222). Recombinant antibodies produced in CHO cells have a normal carbohydrate content, including high fucose, whereas antibodies produced in myeloma cells have low fucose (219). Owing to host cell-dependent fucosylation of the MAb, an antibody produced in myeloma cells may have a 50- to 100-fold higher effector function than the same MAb produced in CHO cells (219, 223). Administration of MAbs in mice that are derived from myeloma cells, such as the RI7-217 antibody (224), are expected to have low fucose and high effector function. CHO cells are the preferred host cell for MAb production, and TfRMAb's produced in CHO cells exhibit no FIRs in mice (225).

### Monoclonal Antibody vs. Peptide Trojan Horses

**Figure 7:**
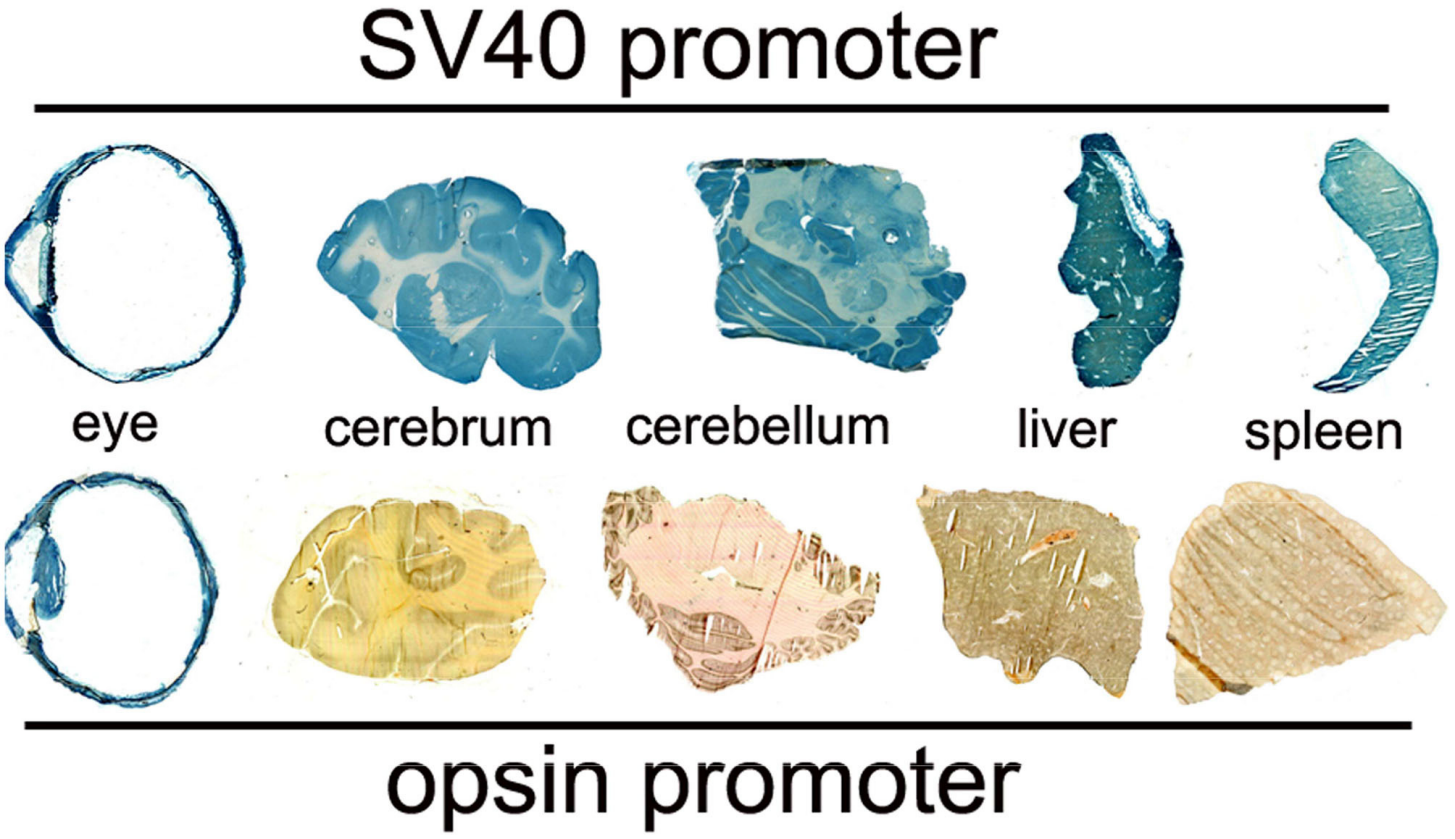
X-gal histochemistry of Rhesus monkey organs removed 2 days after the IV administration of HIRMAb-targeted THLs encapsulated a LacZ β-galactosidase expression plasmid. The LacZ gene was driven either by the widely expressed SV40 promoter (top panel) or the eye-specific opsin promoter (bottom panel). The dose of THL encapsulated plasmid DNA for either monkey study was 70 μg plasmid DNA per monkey. Both monkeys were 6 kg female monkeys. Reproduced with permission from Pardridge (226). The replacement of the SV40 promoter with the opsin promoter eliminates LacZ gene expression in all organs, except for the eye.

Monoclonal antibodies (MAb) may be more robust BBB Trojan horses as compared to peptides. The global delivery of plasmid DNA to brain in the mouse with a TfRMAb (Figure 3), or to the brain in the Rhesus monkey with a HIRMAb (Figure 7), or the global delivery of a lysosomal enzyme to the primate brain with a HIRMAb (Figure 6), has not been demonstrated using a peptide-based Trojan horse. The MAb Trojan horses are species specific. For delivery to rats, the OX26 mouse MAb against the rat TfR is used (186). For delivery to mice, the 8D3 rat MAb against the mouse TfR is used (124). For delivery in humans or Old World primates, the 83–14 mouse MAb against the HIR is used, as this HIRMAb cross-reacts with the insulin receptor of Old World monkeys (51). However, the investigator may choose not to develop a MAb-based Trojan horse for practical reasons, which may include the prohibitive cost of purchase of mg quantities of these antibodies from commercial vendors, or the lack of access to the original hybridoma line. An alternative approach is available to the BBB investigator, which is to contract a service laboratory to produce recombinant forms of these antibodies. This is possible because the amino acid sequences of the variable region of the heavy chain (VH) and the variable region of the light chain (VL) have been published for the OX26 antibody (187), for the 83–14 antibody (181), and for the 8D3 antibody (188). For the heavy chain (HC), the amino acid (AA) sequence of the VH region is fused to the AA sequence of the constant (C)-region. The C-regions of IgG isotypes vary greatly in effector function, e.g., activation of antibody dependent cytotoxicity (ADCC) and C1q complement binding. For the development of antibody therapeutics for cancer, then high effector function is desired. However, for the development of BBB Trojan horse antibodies, low effector function is required. For the engineering of antibodies to be used in mice, the C-region of the mouse IgG1 isotype, which has low effector function, can be used, as described previously (188). The AA sequence of the C-region of the mouse IgG1 HC corresponds to AA 134-457 of the AAB06744 Genbank sequence. For the light chain (LC), the amino acid sequence of the VL is typically fused to the C-region of the mouse kappa light chain C-region, which corresponds to AA 114-219 of the CAA85724 Genbank sequence. The amino terminal sequence of either the HC or the LC should contain 19-20 AA signal peptides, and the HC and LC signal peptide sequences can be suggested by the contract laboratory. From the HC and LC amino acid sequences, the contract laboratory can engineer HC and LC expression plasmids, and eukaryotic host cells, such as Chinese hamster ovary (CHO) cells, can be dual electroporated with the HC and LC expression plasmids. The stably integrated CHO cells may then be grown in small bioreactors in serum free medium followed by protein A affinity chromatography of the recombinant MAb. At a reasonable cost, the contract lab can provide 1–3 g of protein A purified recombinant antibody, with high purity and low endotoxin, as described recently for production of a recombinant form of the 8D3 antibody to the mouse TfR (54), or the 83–14 antibody to the human or primate insulin receptor (53). These amounts of recombinant antibody, if stored properly, can support several years of research with the MAb-based BBB Trojan horse.

The design of recombinant monoclonal antibodies for BBB delivery requires the consideration of multiple parameters including the valency of the antibody, the receptor affinity of the antibody, and the properties of the constant region of the antibody. The constant region can bind to the FcγR, to activate effector function, or the FcRn, to mediate reverse transcytosis from brain back to blood (227). Binding to the FcγR and the FcRn are mediated via different domains of the constant region, and it is possible to produce an aglycosylated form of the antibody that no longer binds FcγR, but still binds the FcRn (218).

### Summary of Brain Receptors Targeted by Trojan Horses: Endothelial vs. Non-endothelial Expression

**Figure 8:**
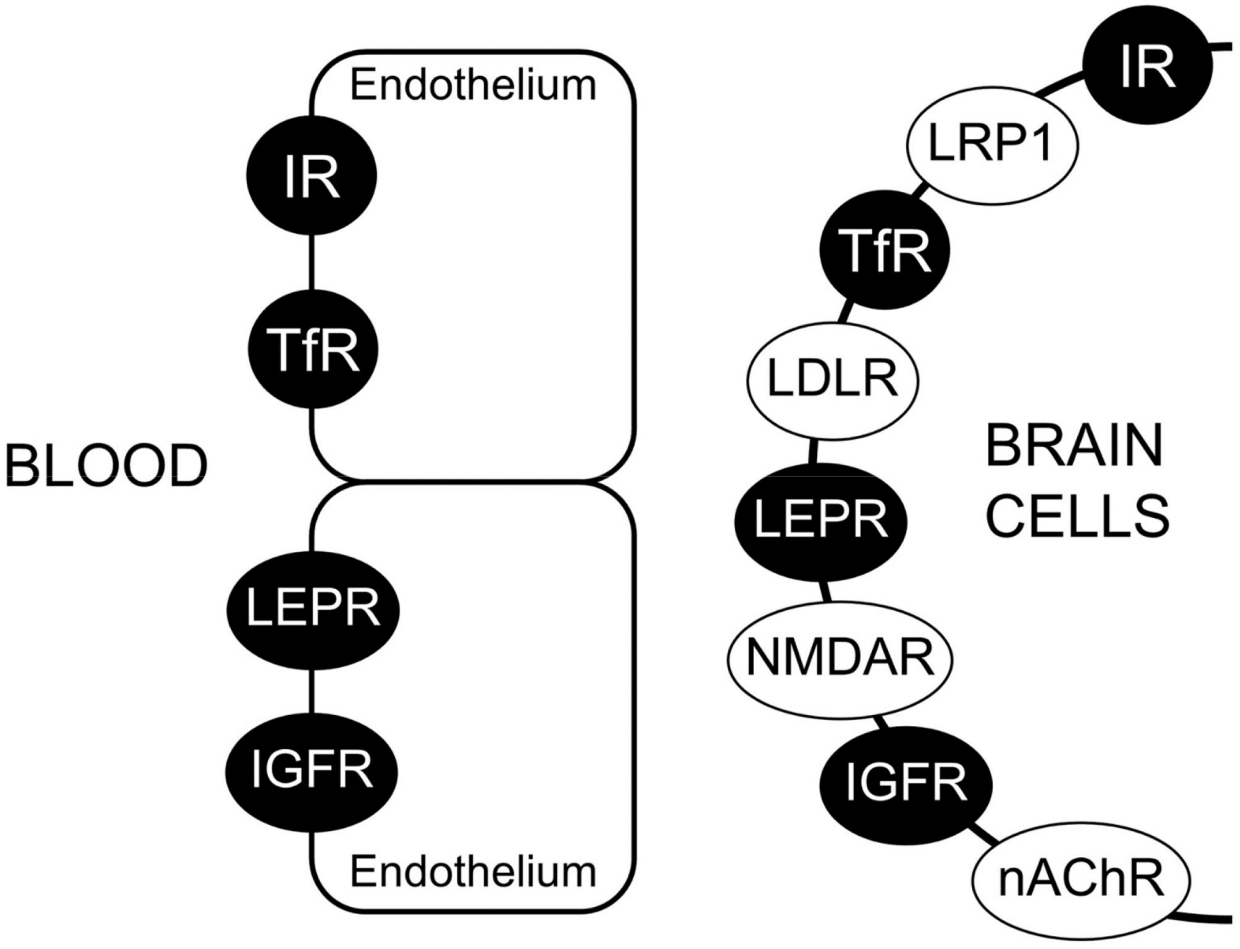
Brain receptors targeted with either peptide or MAb Trojan horses are expressed on either the brain capillary endothelium or on brain cells. Immunohistochemical (IHC) studies show expression on the brain endothelium of the insulin receptor (IR) (51, 98), the transferrin receptor (TfR) (109, 185), the leptin receptor (LEPR) (Figure 4A, (97)), and the insulin-like growth factor receptor (IGFR) (228, 229). However, IHC studies show expression on brain cells beyond the BBB (e.g., pericytes, astrocytes, neurons), but not on brain endothelium, for the low density lipoprotein receptor (LDLR) (98), for the LDL related protein type 1 (LRP1) (115, 116, 118, 119), for the N-methyl D-aspartate receptor (NMDAR) (121), and for the nicotinic acetyl choline receptor (nAChR) (123). IHC shows the IR is also expressed on neurons and astrocytes (230); the TfR is expressed on neurons (231); the LEPR is expressed on neurons and astrocytes (232, 233), and the IGFR is expressed on neurons and astrocytes (228).

Receptor-mediated transport of either a peptide or a MAb Trojan horse across the BBB requires the target receptor be expressed on the brain capillary endothelial membrane. If the target receptor is not expressed on the brain capillary endothelium, then the Trojan horse cannot engage the receptor to trigger transport through the BBB, which is formed by the endothelial cells. The expression of target receptors on the endothelium, as opposed to brain cells, e.g., pericytes, astrocytes, or neurons, which are beyond the BBB, is confirmed by immunohistochemistry (IHC) using light microscopy, electron microscopy, or confocal microscopy. An endothelial receptor produces continuous immune staining of the microvessel, as exemplified in Figure 4A for the leptin receptor (LEPR). Certain BBB receptors are known to be expressed at the endothelium (Figure 8), and these include the insulin receptor (IR), the transferrin receptor type 1 (TfR1), the leptin receptor (LEPR), and the insulin-like growth factor (IGF)-1 receptor (IGF1R), which also has a high affinity for IGF2 (101). Endothelial expression of the IR has been shown with light microscopic IHC in both primate brain (51) and mouse brain (98). Brain endothelial expression of the TfR has been demonstrated with IHC at the light microscopic level (185), the electron microscopic level (109), and with confocal microscopy of isolated rat brain microvessels (110). Brain endothelial expression of the LEPR in either the rat brain or the human brain has been shown by light microscopic IHC (97). Brain endothelial expression of the IGF1R has been shown by confocal microscopy, and co-localization with Factor 8 (229), as well as by pre-embedding electron microscopy (228). In this latter ultrastructural study, the IGF1R was localized only to the endothelial luminal membrane, and not to the endothelial abluminal membrane, because pre-embedding immune staining was performed (228). Brain endothelial abluminal receptors are detected only with post-embedding immune staining (69).

Several other receptors, including the low density lipoprotein (LDL) related protein 1 (LRP1), the LDL receptor (LDLR), the N-methyl D-aspartic acid receptor (NMDAR), which is targeted by glutathione (120), and the nicotinic acetylcholine receptor (nAChR), which is targeted by the RVG peptide (122), are all localized by IHC to brain cells beyond the BBB, such as pericytes, astrocytes, or neurons, without expression of the receptor at the capillary endothelium (Figure 8). IHC of brain with an NMDAR antibody shows expression of this receptor at pericytes, but not endothelium (121). IHC of brain with an antibody to the nAChR shows expression in astrocytes and neurons but not in endothelium (123). IHC with an antibody to the LDLR shows expression in neurons, but not endothelium, whereas this same study shows continuous immune staining of brain microvascular endothelium using an antibody against the IR (98). IHC of brain with a LRP1 antibody shows no overlap with endothelial immune staining using an antibody to the endothelial marker, platelet and endothelial adhesion cell molecule 1 (PECAM1) (115). IHC of brain with a LRP1 antibody shows immune staining in pericytes (117, 118), and astrocytes (119), without endothelial immune staining.

Once a Trojan horse delivers the pharmaceutical cargo across the BBB, owing to Trojan horse engagement of the brain endothelial receptor (Figure 8), the Trojan horse may then deliver the cargo to the intracellular compartment of brain cells, if the same receptor targeted at the endothelium is also expressed on brain cells, as depicted in Figure 8. The insulin receptor is expressed on both neurons and astrocytes (230). The IGFR is expressed on the cell membranes of both astrocytes and neurons (228). The LEPR is expressed on astrocytes and neurons (232, 233). The transferrin receptor is expressed on neurons (231). Therefore, a MAb Trojan horse that engages the IR, the TfR1, the LEPR, or the IGFR, may deliver the cargo across both the BBB and the brain cell membrane (Figure 8).

## Plasmid DNA Engineering

### Antibiotic Resistance-Free (AF) Plasmid DNA for Human Therapeutics

Plasmid DNA are propagated in bacterial hosts in the presence of a selection agent, which is typically the antibiotic, ampicillin. The plasmid DNA vector backbone includes the ampR expression cassette, which encodes for beta lactamase, which degrades ampicillin and confers ampicillin resistance to the host transfected with the plasmid. However, the FDA and the European Medicines Agency (EMA), bar the administration of plasmid DNA to humans which contains such antibiotic resistance genes, and alternative approaches to bacterial clone selection must be adopted (234). Antibiotic resistance free (AF) plasmid can be engineered to allow for antibiotic-free selection. In one such approach, the antibiotic resistance gene is replaced with a 150 base pair RNA-OUT antisense RNA, which represses expression of the levansucrase (SacB) gene, located in the host chromosome (235). The SacB gene converts sucrose to a toxic metabolite, and this allows for selection of transformants with an antibiotic free sucrose medium. High yields of plasmid DNA can be obtained with this system (236), as discussed below in Manufacturing.

### Tissue-Specific Gene Promoters

If the target disease is a lysosomal storage disorder that affects virtually all cells in the body, then a widely expressed promoter can be inserted in the plasmid DNA 5′ to the therapeutic gene. With AAV gene therapy, there is a size restriction to the expression cassette that can be engineered within the AAV DNA, which is <2.3 kb for scAAV and <4.7 kb for ss AAV (9). This size restriction on the expression cassette, which includes the promoter, the therapeutic gene, and the 3′-untranslated region (UTR), restricts the size of the promoter, or the therapeutic gene, that can be used for AAV gene therapy. In contrast, plasmid DNAs as large as 22 kb have been encapsulated in THLs, and expressed in brain *in vivo* (68). Tissue specific promoters as large as the 8 kb tyrosine hydroxylase (TH) promoter have been used in plasmid DNAs encapsulated in THLs for *in vivo* gene expression in brain (68). Owing to the ability to encapsulate large size plasmid DNAs in THL nanomedicines, the therapeutic gene need not be a cDNA form of the gene, but may include chromosomal derived genes, such as the 7.3 kb TH coding region, which is comprised of 13 exons and 12 introns (68). The ability to encapsulate large size plasmid DNA in THLs enables the genetic engineering of plasmid DNA with tissue specific gene promoters, which would be too large to use in AAV gene therapy.

#### Opsin Promoter

Opsin genes encode light sensitive membrane bound G protein coupled receptors, which are largely expressed only in ocular structures such as the retina. A LacZ beta galactosidase expression plasmid under the influence of either the widely expressed SV40 promoter, or the eye-specific opsin promoter, was encapsulated in HIRMAb targeted THLs and injected IV in the Rhesus monkey at a plasmid DNA dose of 12 μg/kg. The brain, eye, and peripheral organs were removed 2 days later, and LacZ gene expression was measured with X-Gal histochemistry (50). The SV40-LacZ transgene was widely expressed in the brain (cerebrum, cerebellum), as well as in the primate eye, liver, and spleen (Figure 7, top panel). The SV40 promoter was replaced with the 2.2 kb bovine rhodopsin (RHO) promoter (237), and this RHO-LacZ plasmid was encapsulated in HIRMAb-targeted THLs, followed by IV injection in the Rhesus monkey. The tissue-specific pattern of LacZ gene expression was very different with the SV40 and opsin promoters (238). The LacZ gene, under the influence of the opsin promoter, was expressed widely throughout the primate eye, but there was no LacZ gene expression in cerebrum, cerebellum, liver, or spleen (Figure 7, bottom panel). Gene expression was observed in the ocular structures other than the retina, owing to expression of the opsin (RHO) promoter in extra-retinal ocular structures (239).

#### GFAP Promoter

Glial fibrillary acidic protein (GFAP) is a cytoskeleton protein expressed in brain with minimal expression in peripheral organs. Brain-specific gene expression of the LacZ reporter gene was tested with the GFAP promoter. Initially, the LacZ gene was placed under the influence of the widely expressed SV40 promoter, and this SV40-LacZ plasmid was encapsulated in 8D3 TfRMAb-targeted THLs and injected IV in the mouse (48). X-gal histochemistry of tissues removed 2 days later showed global expression of the LacZ gene in brain, but also showed LacZ expression in peripheral tissues, such as spleen. When the SV40 promoter was replaced with a 2.2 kb human GFAP promoter to produce a GFAP-LacZ plasmid, the LacZ expression was observed only in brain, with no expression in peripheral tissues, including liver, spleen, heart, and lung (48). The IV injection of GFAP-LacZ expression plasmid encapsulated in THLs targeted with a non-specific IgG resulted in no LacZ expression in any organ including brain, which shows that THL-mediated gene expression is solely a function of the targeting MAb, as well as the gene promoter (48).

In addition to reporter genes, such as LacZ, the GFAP promoter also enabled brain-specific expression of a therapeutic gene encoding tyrosine hydroxylase (TH). TH is the rate-limiting enzyme in dopamine production in brain, which is depleted in an experimental Parkinson's disease (PD) model. The rat TH cDNA was placed under the influence of the 2 kb human GFAP promoter, and this GFAP-TH plasmid DNA was encapsulated in THLs targeted with the OX26 TfRMAb for IV administration to rats. Experimental PD in rats was induced with a unilateral injection of the neurotoxin, 6-hydroxydopamine, in the median forebrain bundle on one side of the brain (240). This model of neurotoxin-induced PD produced a 90% depletion of both TH enzyme activity and immunoreactive TH in the striatum ipsilateral to the injection. Treatment of the PD rats with the THLs encapsulating the GFAP-TH expression plasmid caused a normalization of both TH enzyme activity and immunoreactive TH in the nerve endings of the striatum. In this setting, the GFAP promoter enabled neuronal expression of the TH transgene in the brain (241). These findings are consistent with prior work which showed the 5′-flanking sequence (FS) of the GFAP gene promoter enables neuronal expression (242–244). Astrocyte-specific gene expression requires coordinate interaction between the 5′-FS and the 3′FS of the GFAP gene (243). In addition to enabling TH expression in neurons in brain, the GFAP promoter eliminated off-target effects in peripheral tissues. Treatment of PD rats with THLs carrying the SV40-TH gene caused an increase in TH enzyme activity not only in brain, but also in liver. However, no TH expression in liver was observed in the PD rats treated with THLs encapsulating the GFAP-TH plasmid (241).

#### Tyrosine Hydroxylase Promoter

Tyrosine hydroxylase (TH) expression in brain is generally confined to the nigral-striatal tract of brain, which is the primary site of neurodegeneration in PD. The diminished TH gene expression in PD can be increased by either TH gene replacement therapy or by reversing the nigral-striatal neurodegeneration via neurotrophin gene therapy. Since the cause of PD is neurodegeneration in the nigral-striatal tract, neurotrophin gene therapy is a more definitive treatment of PD than is TH enzyme replacement therapy. A potent neurotrophic factor for the nigral-striatal tract is glial derived neurotrophic factor (GDNF) (245). The goal of GDNF gene therapy in PD is to produce selective expression of the GDNF transgene in the nigral-striatal tract of brain, and not in other sites of brain that may produce off-target effects. To insure TH gene expression only in the nigral-striatal tract of brain, a cDNA encoding human preproGDNF (P39905) was engineered with 8 kb of the rat TH gene promoter (pro), and this THpro-preproGDNF expression plasmid was encapsulated in THLs targeted with the rat OX26 TfRMAb, and administered IV to rats with toxin-induced experimental PD (246). Confocal microscopy of the THL treated PD rats showed expression of the GDNF transgene in the substantia nigra ipsilateral to the toxin injection. In addition, this treatment caused an increase in TH enzyme activity and an increase in immunoreactive TH in the striatum ipsilateral to the toxin injection. The rats were treated with THLs encapsulating the THpro-GDNF with a single injection at 2 weeks after the unilateral neurotoxin injection in brain, and were then euthanized 6 weeks after this THL injection. The study showed a lasting neurotrophic effect of GDNF gene therapy, as TH activity in the lesioned striatum remained high at 4–6 weeks following 1–3 administrations of THLs following neurotoxin administration (246, 247).

#### Platelet Derived Growth Factor B (PDGFB) Promoter

Niemann Pick C1 (NPC1) is a severe neurodegenerative disorder caused by mutations in the NPC1 gene (248). For NPC1 gene therapy of the brain, it is desirable to use a neuron-specific gene promoter, such as the 5′-flanking sequence (FS) of the human platelet derived growth factor B chain (PDGFB). Prior work showed neuronal expression in brain is conferred by the region from nucleotides −1,360 to +75, relative to the transcription start site, of the human PDGFB promoter (249). This nucleotide sequence of the PDGFB promoter was derived by Blast alignment of the human PDGFB gene (NG_002599) and the human PDGFB mRNA (NM_002608). A NPC1 expression plasmid was engineered where the 1.5 kb human PDGFB promoter was placed 5′ of the 4.0 kb human NPC1 open reading frame, which corresponds to nucleotides 164-4000 of NM_000271 (54). This PDGFB-NPC1 transgene was encapsulated in THLs targeted with the 8D3 TfRMAb for the treatment of the NPC1 null mouse, as discussed below.

## Target Diseases of the CNS

### Orphan Diseases of Brain

Orphan diseases of the brain include rare disorders that affect <200,000 individuals in the US, and such diseases are invariably inborn errors of metabolism. Of the 7,000 rare diseases that affect 25 million subjects in the US, about one-third, or >2,000 diseases, affect the CNS. Many of these CNS orphan diseases could be treated with gene therapy. Indeed, the only AAV-based gene therapies currently approved by the FDA include Luxturna, for a rare form of blindness (250), and Zolgensma, for infantile spinal muscular atrophy (10), both orphan diseases. Both are approved only for single-dose treatments. Luxturna is administered by sub-retinal injection in each eye, and Zolgensma is administered as a single IV injection. Other orphan diseases of the brain that are treatable with gene therapy include the wide array of genetic diseases, which includes lysosomal storage disorders, such as the Mucopolysaccharidoses (MPS) and NPC1, or inherited forms of blindness.

#### Mucopolysaccharidoses

MPS Type VII (MPSVII) is caused by mutations in the gene encoding the lysosomal enzyme, beta-glucuronidase (GUSB). Gene therapy of the MPSVII GUSB^−/−^ null mouse was evaluated by engineering a human GUSB expression plasmid DNA driven by the cytomegalovirus (CMV) promoter, with a bovine growth hormone (BGH) polyA termination sequence (251). This plasmid DNA, designated pCMV-GUSB was encapsulated in THLs targeted with the 8D3 TfRMAb, and GUSB gene expression was assessed by determination of GUSB enzyme activity using a fluorometric enzymatic assay. TfRMAb/pCMV-GUSB THLs were added to GUSB^−/−^ null fibroblasts, and both a dose response and a time response study was performed in cell culture. In untreated fibroblasts, the intracellular GUSB activity was <1 unit/mg protein, where 1 unit=1 nmol/hr. In fibroblasts treated with a single addition of 4 μg plasmid DNA per dish encapsulated in TfRMAb targeted THLs at day 1, the intracellular GUSB was still high, 48 ±1 units/mg protein, at 16 days after the single application of THLs on day 1. In the dose response study, THL encapsulated DNA was added at 0.4, 1.0, 4.0, and 10 μg plasmid DNA/dish, and the intracellular GUSB enzyme activity was 2.9 ± 0.1, 6.7 ± 0.4, 22.4 ± 0.5, and 37.2 ± 1.4 units/mg protein, respectively, after a 48 h incubation of the null fibroblasts with the THLs (251). For the *in vivo* gene therapy study, GUSB null mice were separated into two treatment groups and treated with either saline or with 10 ug/mouse of pCMV–GUSB plasmid DNA encapsulated in 8D3-targeted THLs. Mice were sacrificed at 48 h after the single IV administration and brain and other organs were removed for measurement of GUSB enzymatic activity. The GUSB enzyme activity in the saline treated animals was <0.2 units/mg protein in brain and all other organs. The GUSB enzyme activity in brain, liver, spleen, lung, and kidney was increased more than 10-fold at 2 days following a single IV injection of the THLs. There was no increase in GUSB enzyme activity in the heart, indicating THLs do not cross the myocardial endothelial barrier, owing to the lack of endothelial TfR expression in heart. GUSB enzyme activity in serum in the mice was <2 and 10.3 ± 0.1 U/ml in the saline and THL treated mice, respectively, at 48 h after THL injection. These organ levels of GUSB enzyme activity produced by THL administration are sufficient to reverse the course of lysosomal storage pathology (252).

#### Niemann Pick Disease Type C1 (NPC1)

NPC1^−/−^ null mice (253, 254) were treated with the pPDGFB-NPC1 plasmid DNA described above, which was encapsulated in THLs targeted with a recombinant form of the 8D3 TfRMAb (54). A cohort of 36 null mice were treated weekly with IV injections, and 18 mice were treated with saline and 18 mice were treated with the TfRMAb/pPDGFB-NPC1 THLs starting at 6–7 weeks and ending at week 11. The mice were euthanized 4 days following the 11th week injections, and brain, liver, and spleen harvested for quantitative PCR, for reverse transcriptase (RT) PCR, and for organ histology. PCR primers specific for the human NPC1 mRNA (NM_000271), were designed which did not cross react with mouse NPC1 mRNA (AF003348). The real time PCR of the tissue samples was performed in parallel with a pPDGF-NPC1 plasmid DNA standard curve, so the PCR parameter, Cq (earliest cycle of fluorescence detection), could be converted to pg plasmid DNA/mg wet tissue delivered to the mouse brain, liver, and spleen. There is no measurable pPDGF-NPC1 plasmid DNA in the organs of the saline treated mice, and high levels of pPDGF-NPC1 plasmid DNA were detected in organs from mice treated with THLs in the rank order of spleen>liver>brain (Table 1). The brain plasmid DNA concentration is 10 pg DNA per mg tissue (Table 1), which corresponds to 1.1 × 10^6^ DNA molecules per mg brain tissue, based on the molecular weight of a 8.0 kb double stranded plasmid DNA of 5.3 × 10^6^ g/mole. There are 100 million cells per 400 mg mouse brain (255), or 0.25 × 10^6^ cells/mg brain. These calculations indicate there are 4–5 plasmid DNA molecules delivered to every cell in brain of the mouse with the THL delivery system, which correlates with prior estimates of THL delivery of plasmid DNA to brain cells in the primate (256). RT-PCR was used to determine the level of NPC1 mRNA produced in the tissues following transcription of the THL-delivered pPDGFB-NPC1 DNA (Table 2). In parallel with RT-PCR of the NPC1 mRNA, RT-PCR was also performed for a housekeeping gene, glyceraldehyde 3′-phosphate dehydrogenase (GAPDH). The ΔCq is the difference between the Cq for NPC1 and the Cq for GAPDH for a given organ. The ΔΔCq is the difference between the ΔCq for the saline treated mouse vs. the THL treated mouse. The change in NPC1 mRNA with THL gene therapy was computed from the base 2 antilog of the ΔΔCq parameter for each organ (Table 2). THL therapy increased the NPC1 mRNA, relative to the GAPDH mRNA, by 8,192-fold, 338-fold, and 238-fold in spleen, brain, and liver, respectively. THL treatment of the NPC1 mice caused visible reductions in lysosomal inclusion bodies in brain, liver, and spleen, and decreased astrogliosis in cerebrum. However, starting THL gene therapy at 6–7 weeks of age in the NPC1 mouse did not prolong survival (54). This indicates disease stage at the start of gene therapy determines the therapeutic outcome, as observed for other lysosomal storage disorders (257). When THL treatment of the NPC1 mice was initiated at weeks 6–7, these NPC1 mice already have a severe accumulation of autophagic lysosomal inclusion bodies, diffuse astrogliosis, and suppression of myelin formation (258–260). In future treatment of the NPC1 mouse, the THL gene therapy needs to be started immediately after birth before there is significant formation of cholesterol laden lysosomal inclusion bodies.

**Table 1:** Organ concentrations (pg plasmid DNA per mg wet tissue) in female and male NPC1 mice at 4 days following the last IV injection of either vehicle or TfRMAb targeted THLs encapsulated with the pPDGFB-NPC1 expression plasmid DNA.

**Sex**	**Treatment**	**Brain**	**Spleen**	**Liver**
Female	Vehicle	<0.6	<0.6	<0.6
Male	Vehicle	<0.6	<0.6	<0.6
Female	THL	10.1 ± 3.1	107.3 ± 40.5	39.9 ± 7.9
Male	THL	8.7 ± 0.6	58.6 ± 12.9	30.0 ± 7.8

*Mean ± SEM (N=4 male or 4 female mice per vehicle or THL treatment group). PCR Cq values were converted to pg plasmid DNA with a pPDGF-NPC1 plasmid DNA standard curve. Reproduced with permission of Jiang et al. (54). NPC1, Niemann Pick type C1*.

**Table 2:** Organ enrichment of NPC1 mRNA, relative to GAPDH mRNA, in THL treated NPC1 mice as compared to vehicle treated NPC1 mice.

**Organ**	**Treatment**	**NPC1 Cq**	**GAPDH Cq**	**ΔCq**	**ΔΔCq**	**NPC1 mRNA fold change**
Spleen	Vehicle	35.5 ± 2.6	18.8 ± 1.1	16.9 ± 2.1	–	–
Spleen	THL	22.8 ± 0.7	18.9 ± 0.4	3.9 ± 1.0	13.0	8,192
Liver	Vehicle	33.9 ± 2.8	24.2 ± 1.7	10.0 ± 2.6	–	–
Liver	THL	26.4 ± 0.9	24.3 ± 1.3	2.1 ± 1.0	7.9	238
Brain	Vehicle	37.0 ± 1.4	21.4 ± 0.4	15.9 ± 1.2	–	–
Brain	THL	29.6 ± 0.8	22.1 ± 1.6	7.5 ± 1.8	8.4	338

*ΔCq = (NPC1 Cq – GAPDH Cq); ΔΔCq = [ΔCq (vehicle mice)—ΔCq (THL mice)]; NPC1 mRNA fold change between vehicle mice and THL mice computed from base two anti-log of ΔΔCq. Cq values are mean ± SD [eight mice (four males; four females)] in vehicle group; 8 mice [(4 males; 4 females)] in THL group. Reproduced with permission of Jiang et al. (54). GAPDH, glyceraldehyde 3′-phosphate dehydrogenase*.

#### Blindness

**Figure 9:**
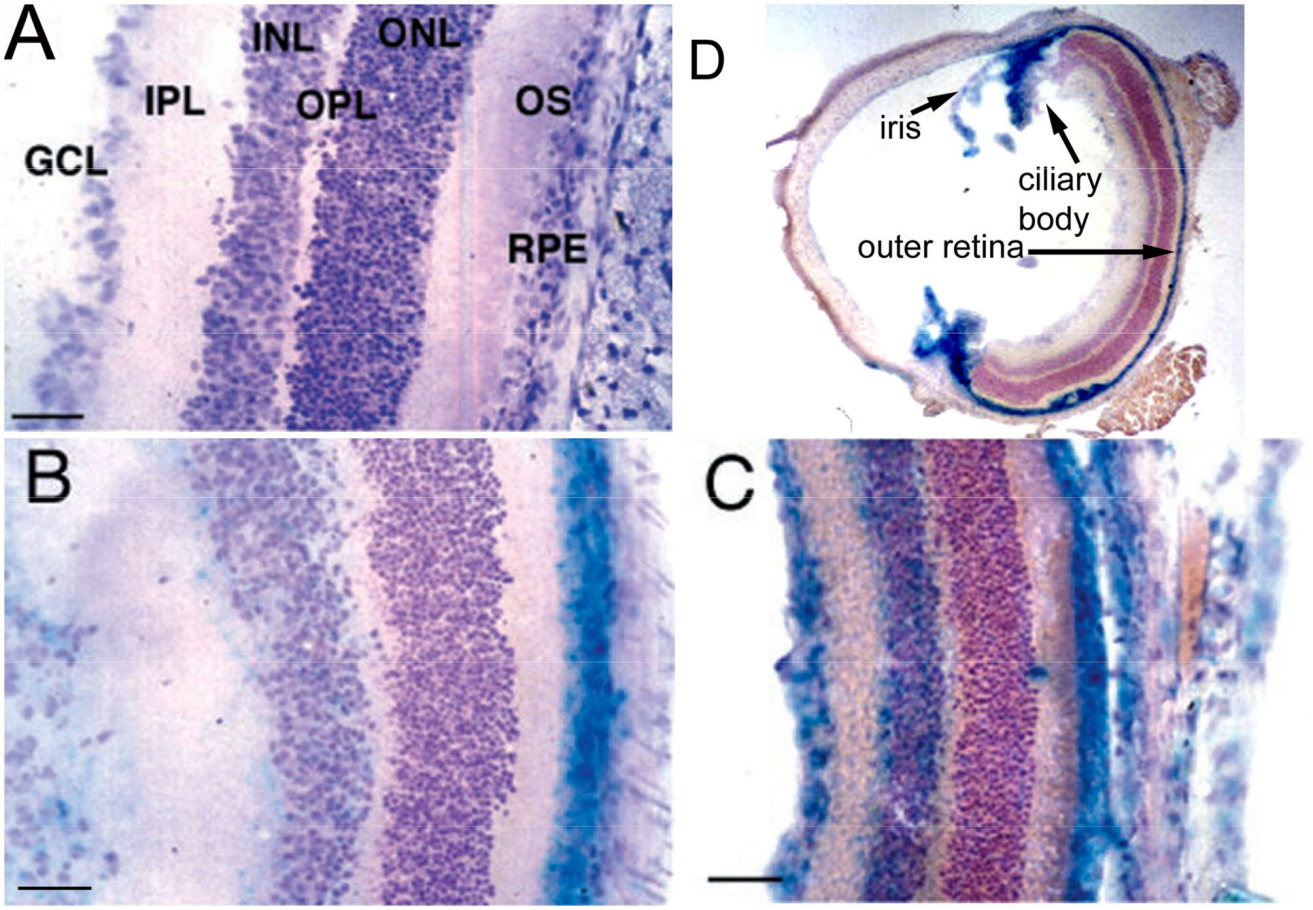
β-galactosidase histochemistry of the mouse eye removed 48 h after the IV administration of the THLs targeted with the 8D3 MAb against the mouse TfR and encapsulating a LacZ β-galactosidase expression plasmid DNA under the influence of either the SV40 promoter **(B,D)** or the GFAP promoter **(C)**. **(A)** Eye of a control, uninjected mouse counter-stained with Mayer's hematoxylin. **(B)** The retina of a mouse injected with the SV40/ β-galactosidase plasmid encapsulated within the 8D3-targeted THL shows gene expression restricted to the RPE of the outer retina, with minimal gene expression in the inner retina or GCL. **(C)** An eye of a mouse injected with the GFAP/ β -galactosidase expression plasmid encapsulated within the 8D3-targeted THL, shows gene expression in the RPE of the outer retina, the GCL of the inner retina, and at the border of the IPL and the INL. **(D)** β -Galactosidase histochemistry in the mouse eye obtained 48 h after IV injection of the SV40/β-galactosidase plasmid encapsulated within the THL targeted with the 8D3 MAb. There is diffuse expression of the transgene in the RPE of the outer retina, as well as in the iris and the ciliary body. Reproduced with permission from Zhu et al. (261). GCL, granule cell layer; IPL, inner plexiform layer; INL, inner nuclear layer; OPL, outer plexiform layer; ONL, outer nuclear layer; OS, outer segments; RPE, retinal pigmented epithelium. Magnification bars in panels **A** and **B**, 40 microns; magnification bar in **(C)**, 60 microns.

The outer retina is comprised of the retinal pigmented epithelium (RPE), which is a high resistance epithelial barrier that provides nutrients to the projections of the rods and cones in the outer segment (OS) of the retina (Figure 9A). The cell bodies of the rods and cones are in the outer nuclear layer (ONL), whereas cell bodies of amacrine, horizontal, and bipolar cells are in the inner nuclear layer (INL) (Figure 9A). There are >50 clinical trials testing AAV gene therapy of blindness (250). AAV gene therapy of the outer retina involves bilateral subretinal injections, and transgene expression is confined to retinal cells near the needle injection site (262). A broader distribution of transgene expression in the retina may occur if the injection causes a retinal detachment (263). Non-invasive global distribution of a therapeutic gene to the retina is possible with a trans-vascular approach using THLs. Following IV administration of THLs, the gene traverses the retinal microvasculature, which along with the RPE, forms the blood-retinal barrier (BRB). The global delivery of a transgene to both the inner and outer retina with THLs in the mouse is shown in Figure 9. A LacZ expression plasmid under the influence of either the SV40 promoter or the GFAP promoter was encapsulated in THLs targeted with the 8D3 TfRMAb, and injected IV in the mouse at a dose of 5 ug/mouse of THL encapsulated DNA (261). The eyes were removed 2 days later followed by X-Gal histochemistry (Figure 9). Following administration of the SV40-LacZ plasmid, the transgene was expressed primarily in the RPE of the outer retina (Figure 9B). However, following administration of the GFAP-LacZ plasmid, the transgene was expressed in both the RPE of the outer retina as well as the INL and granule cell layer (GCL) of the inner retina (Figure 9C). The transgene was also expressed in the iris and ciliary body following administration of either the SV40-LacZ (Figure 9D) or the GFAP-LacZ (261). Gene expression in the extra-retinal structures of the eye such as the iris or ciliary body is consistent with expression of the TfR in these structures (264, 265). Expression of the GFAP-LacZ in the iris and ciliary body is also consistent with the known GFAP expression in these regions of the eye (266, 267). There is a broader retinal expression profile of the LacZ in the Rhesus monkey retina following the IV injection of HIRMAb-targeted THLs encapsulating either the SV40-LacZ plasmid DNA or the opsin-LacZ plasmid DNA [Figure 7, (238)]. With either promoter, the LacZ gene is widely expressed in both the inner and outer retina of the primate. A therapeutic gene can be selectively targeted to a given region of the retina with THLs that encapsulate a plasmid DNA with a tissue-specific promoter, such as opsin (Figure 7). The broader ocular expression of the LacZ gene in the Rhesus monkey, treated with HIRMAb targeted THLs (Figure 7), as compared to the ocular expression of the LacZ gene in the mouse, treated with TfRMAb targeted THLs (Figure 9), may be due to the broad expression of the insulin receptor gene in multiple structures of the human eye (268).

### Non-orphan Diseases of the Brain

#### Brain Cancer

Glioblastome multiforme (GBM) is the most malignant form of primary brain cancer. BBB delivery systems are needed for the treatment of GBM, because the BBB within the tumor is intact, except in the necrotic core of large tumors (269, 270). New nanomedicine therapeutics for the treatment of brain cancer are being developed. A lipid nanoparticle was complexed to oligonucleotides against the micro RNA-21 (miR-21). The nanoparticles were targeted to glial cells with chlorotoxin (CTX), a 36-amino acid snake venom peptide that binds matrix metalloproteinase (MMP)-2 on glial cells (271). No Trojan horse was incorporated in the nanomedicine to circumvent the BBB within the tumor.

Over-expression of the EGF receptor (EGFR) is found in 90% of GBM, and this receptor plays an oncogenic role in brain cancer (272). THLs doubly targeted with both a HIRMAb and a MAb against the mouse TfR were prepared for the treatment of intracranial GBM brain cancer in scid mice (58, 59). Human U87 glioma cells were implanted in the caudate-putamen nucleus (CPN) on one side of the brain of the mice. By 5 days, the tumor had grown to fill the CPN volume. THLs were administered weekly by IV injection starting at day 5 after tumor implantation. These THLs were targeted with the HIRMAb to trigger receptor-mediated endocytosis into the human glioma cell beyond the blood-tumor barrier. However, this HIRMAb does not recognize the murine IR on the vasculature perfusing the blood tumor barrier, which arises from the microvasculature of contiguous mouse brain. Therefore, the THLs were also targeted with the 8D3 MAb against the mouse TfR to enable transport of the THL across the mouse tumor vasculature. Over-expression of EGFR in this tumor was shown by immunocytochemistry (58). Expression of the HIR within the tumor cells and expression of the murine TfR within the mouse brain parenchyma and within the tumor capillaries, which were of mouse brain origin, were also demonstrated by immunocytochemistry (59). The goal was to prolong survival time with gene therapy aimed at knocking down the tumor EGFR mRNA. In one study, the THLs encapsulated an expression plasmid encoding a 700 nucleotide (nt) antisense RNA directed against nt 2317-3006 of the human EGFR mRNA (X00588). Mice were treated weekly with (a) saline, (b) 5 μg/week of THL encapsulated anti-EGFR plasmid DNA, or (c) 5 μg/week of THL encapsulated with a luciferase expression plasmid DNA as a negative control. Treatment with the EGFR antisense gene caused a 100% increase in survival time over the saline treated mice (58). The survival of the mice treated with doubly targeted THLs encapsulating a luciferase expression plasmid DNA was no different than the survival of mice treated with saline (58). In a second study, RNA interference (RNAi) was tested with a plasmid DNA that encoded a short interfering RNA (shRNA) targeting nt 2529-2557 of the human EGFR mRNA, under the influence of the U6 RNA polymerase promoter (59). Treatment of U87 cells in culture with the HIRMAb-targeted THLs encapsulating the plasmid DNA encoding the anti-EGFR shRNA caused a 90% inhibition of EGF-mediated release of cytosolic calcium (59). The THLs encapsulating the anti-EGFR shRNA expression plasmid DNA were doubly targeted with the HIRMAb and TfRMAb for treatment of the scid mice with the intracranial U87 glial tumor. Weekly THL treatment caused an 88% increase in survival in the tumor bearing mice compared to the mice treated with saline. These combined studies show that DNA-based RNAi, encoding shRNA, is comparable in potency to classical antisense gene therapy in knocking down a target oncogene, such as the EGFR.

#### Parkinson's Disease

Current approaches to the viral gene therapy of PD are aimed at replacement of enzyme activity, e.g., tyrosine hydroxylase (TH), which converts tyrosine to L-DOPA, or aromatic amino acid decarboxylase (AAAD), which converts L-DOPA to dopamine (273). AAV encoding AAAD was injected bilaterally into the left and right putamen of the brains of patients with PD (274). So as to distribute the viral vector to a significant part of the putamen, which has a volume of 3.5 mL in the human brain (275), the injection volume of the AAV-AADC was increased 10-fold to 900 μL per putamen, which produced transgene expression in 21–42% of the putamen (274). However, enzyme replacement therapy does not treat the basic cause of PD, which is neurodegeneration of the nigral-striatal tract. Moreover, owing to the size limitation of the expression cassette that can be cloned into the AAV genome, AAV gene therapy for PD is limited with respect to the design engineering of the promoter/therapeutic gene. This is not the case for THLs, as plasmid DNAs as large as 22 kb have been encapsulated in THLs with *in vivo* gene expression (68). Gene therapy of PD should be directed at halting the neurodegeneration of this disease, and one of the most potent neurotrophic factors for PD is GDNF (245). An expression plasmid DNA was engineered for the treatment of experimental PD in rats, which is induced by the unilateral intra-cerebral injection of the neurotoxin, 6-hydroxydopamine, into the median forebrain bundle on one side of the brain (246). So as to localize GDNF gene expression to the nigra-striatal tract, the human prepro GDNF cDNA was placed under the influence of 8 kb of the rat TH promoter. This 13 kb expression plasmid DNA was encapsulated in THLs targeted with the OX26 MAb against the rat TfR (247). Experimental PD was induced by toxin injection, and weekly IV THL administration was performed at weeks 1, 2, and 3 after toxin injection. Rats were euthanized at 6 weeks after toxin administration. At this time, the aberrant motor activity of experimental PD had been nearly completely eliminated based on apomorphine and amphetamine rotation. The striatal TH enzyme activity was measured at 6 weeks after toxin administration for both the lesioned and non-lesioned sides of brain in the saline and THL treated rats. There was a 99% reduction in striatal TH enzyme activity on the lesioned side in the saline treated animals. However, the reduction in striatal TH enzyme activity was only 23% in the animals treated with THLs at 1, 2, and 3 weeks after toxin injection. These studies show that lasting effects of a short course of GDNF gene therapy is produced in new onset experimental PD with a tissue specific promoter such as the 8 kb TH promoter combined with GDNF neurotrophin gene therapy (247).

#### Alzheimer's Disease

Patients with Alzheimer's disease (AD) develop dementia associated with degenerating neurites near amyloid plaques. Neurotrophins, such as nerve growth factor (NGF), have been proposed as novel treatments to promote neurite development in AD. NGF does not cross the BBB, but a conjugate of NGF and a TfRMAb is able to penetrate the brain and restore cholinergic function in a chemical lesion model in rats (276). In an attempt to increase NGF expression in the brain of subjects with AD, AAV gene therapy was examined in a phase 2 clinical trial of AD subjects (277). The AAV2 serotype was engineered to express human NGF and was injected bilaterally in the nucleus basalis Meynert (nbM) of AD patients. The AAV2-NGF was injected in 20 μL volumes in multiple sites of the nbM. However, the NGF gene therapy produced no improvement in cognitive decline in the AD patients (277). A subsequent post-mortem examination of the NGF distribution in brain in these subjects showed the zone of NGF expression was confined to a small region of 0.96 mm from the intra-cerebral injection site (278). This finding is expected since it is known that viral distribution in brain, following an intra-cerebral injection, is confined to the needle injection site (279). The problem with the NGF clinical trial for AD was not the lack of efficacy of NGF in AD, but rather to the limitations of the brain gene delivery system that injected the AAV into a localized region of brain (278). An alternative approach is the THL delivery of a plasmid DNA across the BBB where this plasmid encodes for human prepro-NGF under the influence of a promoter specific for the nbM, or previously described promoters specific to cholinergic neurons (280).

## Trojan Horse Liposome Manufacturing

### Upstream Manufacturing: Scalable THL Manufacturing

**Figure 10:**
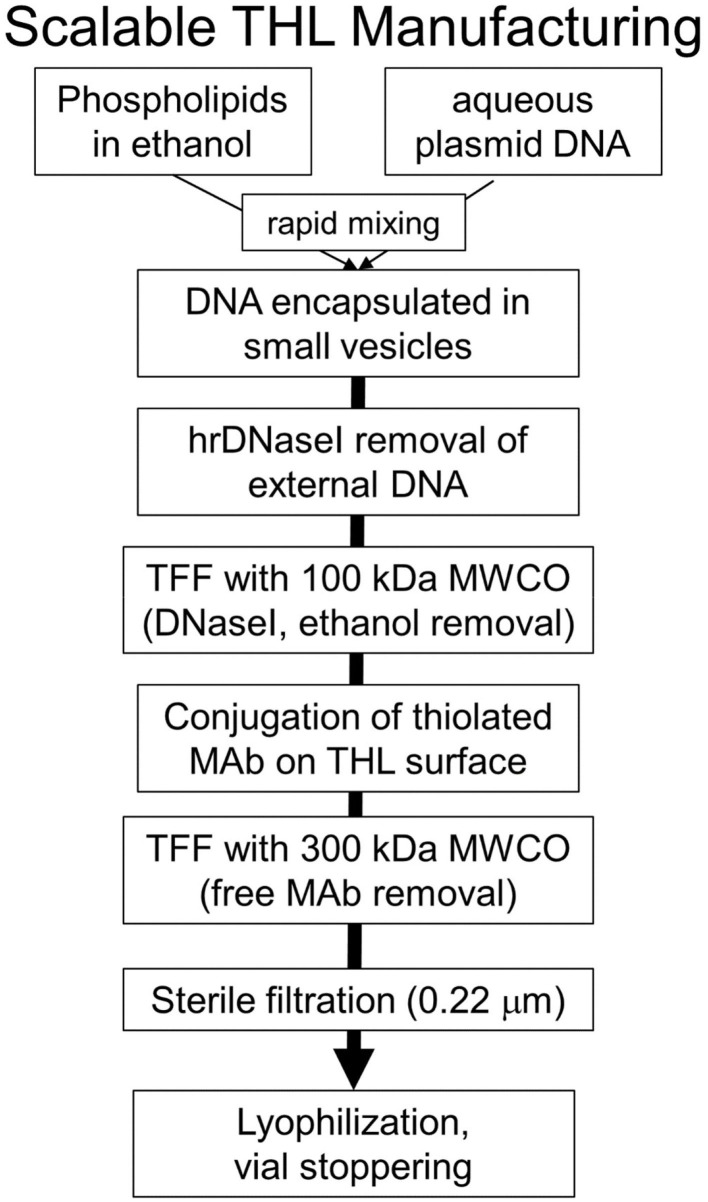
Scalable THL manufacturing using ethanol dilution to encapsulate the plasmid DNA within small 100–150 nm vesicles, and using tangential flow filtration (TFF) with a 100 kDa molecular weight cut-off (MWCO) to remove the nuclease and ethanol, and a second TFF with a 300 kDa MWCO to remove the unconjugated MAb. The final THL formulation is sterile filtered, and lyophilized for long term storage. hrDNaseI, human recombinant DNase I.

The thin film/extrusion method used to prepare THLs for preclinical research includes steps, such as vortexing, sonication, extrusion, and gel filtration (52), which are not scalable for commercial manufacturing. All of these steps can be eliminated with an ethanol dilution manufacturing process outlined in Figure 10. The phospholipid stocks (~25 mM) are prepared in 100% ethanol. This solution is diluted to 90% ethanol and rapidly mixed with an equal volume of 1 mg/mL of the plasmid DNA in aqueous solution, so that the final ethanol is 45%. This ethanol dilution process encapsulates the plasmid DNA in small vesicles (281). THL manufacture with the ethanol dilution method takes advantage of the dual properties of ethanol dilution. First, high ethanol concentrations cause condensation of single DNA molecules into compact structures (282). Without such condensation, the gyration diameter of a 10 kb supercoiled plasmid DNA, which approximates 460 nm (283), would exceed the diameter of a 100–150 nm small lipid vesicle. Second, ethanol dilution promotes the conversion of large multivesiclar liposomes into small 100–150 nm vesicles (284). Following the encapsulation of the plasmid DNA in the interior of small lipid vesicles, the preparation is treated with human recombinant DNaseI, which removes any external plasmid DNA not encapsulated. Next, tangential flow filtration (TFF) with a molecular weight cutoff (MWCO) of 100 kDa removes the ethanol, DNase, and degraded external nucleic acid (Figure 10). The thiolated MAb is then conjugated to the maleimide moiety on the surface PEG, and the unconjugated MAb is removed by TFF with a 300 kDa MWCO. The final step is sterile filtration prior to lyophilization as described below. The ethanol dilution method is scalable to the 5–10 L stage for either small molecules (285) or plasmid DNA (281). However, downstream manufacturing solutions have to be developed to enable long term storage of THL therapeutics for commercial use, and this is enabled by a successful lyophilization process of the THLs.

### Downstream Manufacturing: THL Lyophilization

**Figure 11:**
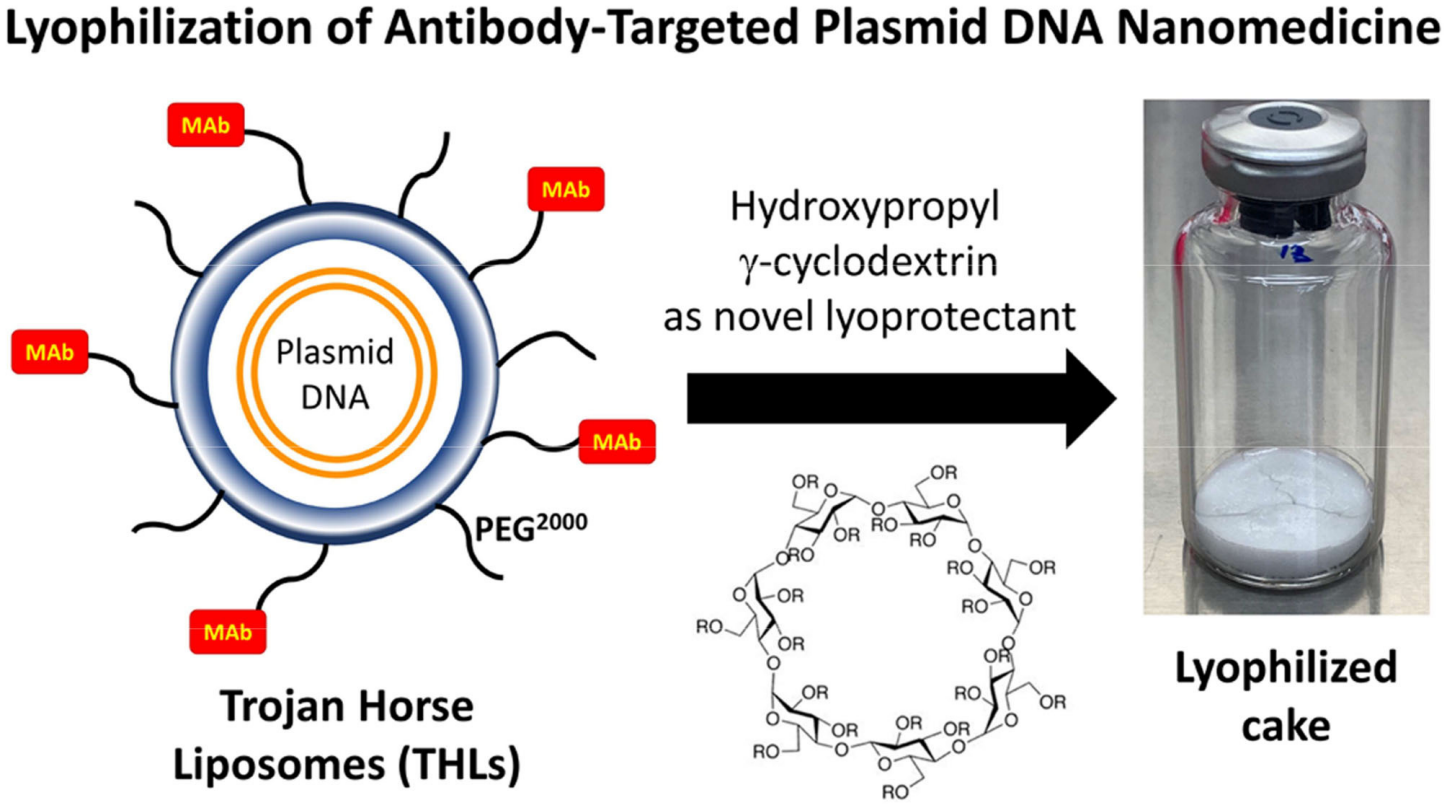
THLs are complex nanomedicines with plasmid DNA encapsulated in the interior of the pegylated liposome, and the tips of the 2000 Da PEG strands are conjugated with the receptor specific monoclonal antibody (MAb). After an iterative process of lyoprotectant optimization, the ideal lyoprotectant for lyophilization of THLs is hydroxypropyl-γ-cyclodextrin (HPγCD) at a 40:1 molar ratio relative to the phospholipid. The lyophilization produces a stable cake without cavitation, which produces a clear solution after hydration. The THLs lyophilized with HPγCD can be stored at 4°C for at least 6 months with minimal loss of gene expression potency. Reproduced with permission from Lee et al. (53).

The long-term storage of THLs as a liquid formulation is not possible as liposomes are unstable when stored as a liquid with either small molecules (286) or with plasmid DNA (287, 288). THL lyophilization could enable long-term storage, providing the freeze-dry process can be optimized to ensure retention of THL potency following lyophilization, storage, and hydration for use. THLs are complex nanomedicines with plasmid DNA in the interior of the pegylated liposome, and MAb Trojan horses tethered to the surface of the THL, and it was not clear if such formulations could be successfully lyophilized, followed by hydration with retention of the original structural and functional properties of the THL. The key parameter in the lyophilization of THLs is optimization of the lyoprotectant. The disaccharide, trehalose, was originally shown to stabilize the encapsulation of a small molecule in freeze-dried liposomes (289), and trehalose has been used for the lyophilization of plasmid DNA lipoplexes (290). However, in the initial evaluation of lyophilization of THLs, it was found that trehalose is unsatisfactory as a THL lyoprotectant. Following hydration of the trehalose lyophilized THLs, the liposomes fused and release the plasmid DNA (53). Therefore, a series of alternative lyoprotectants was evaluated. Anionic polymers such as sulfobutylether-β-cyclodextrin (SBECD) or hyaluronic acid have been used previously as lyoprotectants for small molecules, β-galactosidase, or siRNA polyplexes (291–293). Gamma-cyclodextrin (βCD) has been used as a lyoprotectant for enzymes (294). However, liposome fusion and release of DNA was observed following hydration of THLs freeze dried with SBECD, hyaluronic acid, or γCD (53). Hydroxypropyl-β-cyclodextrin (HPβCD) was found to be superior to trehalose in the freeze dry of liposomes prepared with saturated phospholipids and encapsulating a small molecule (295). However, HPβCD was shown not to be a suitable lyoprotectant when the liposomes were prepared with an unsaturated phospholipid (296). The major lipid comprising THLs is POPC, which is an unsaturated phospholipid. Following an iterative process, it was found that the use of hydroxypropyl-γ-cyclodextrin (HPγCD), at a 40:1 wt:wt ratio of HPγCD:phospholipid as a lyoprotectant, coupled with a programmed process of freezing, primary dry, and secondary dry, produced a freeze-dried THL cake without collapse (Figure 11). The THL cakes were stored at 4°C for up to 6 months. Following hydration, there was no liposome fusion, minimal DNA leakage and retention of gene expression potency that varied from 45 to 100% (53). The Certificate of Analysis (COA) of the freeze-dried THL is shown in Table 3, which gives the results of 14 test methods on the quality, strength, identity, potency, purity, and safety of the hydrated freeze-dried THLs.

**Table 3:** Certificate of analysis of freeze-dried THLs tested in Rhesus monkeys.

**Purpose**	**Parameter**	**Test method**	**Specifications**	**Test result**
Quality	Appearance	Visual inspection	Amorphous cake with clear solution after hydration	Pass
	Water content	Coulometry	<0.2% w/w	0.13% w/w
Strength	DNA	Fluorometry	50–100 μg/vial	81 μg/vial
	HIRMAb	Bicinchoninic acid	150–300 μg/vial	225 μg/vial
	Phospholipid	Enzymatic spectrophotometry	2–3 mg/vial	2.3 mg/vial
	Cholesterol	Enzymatic spectrophotometry	50–100 μg/vial	77 μg/vial
Identity	IgG conjugation	Western blot	Heavy and light chains conform to HIRMAb standard	Pass
	THL diameter	Dynamic light scattering	100–200 nm	135 ± 40 nm
	PDI		<0.3	0.09
Potency	HIR ED50	HIR ELISA	<8 nM	5.1 ± 1.8 nM
	Luciferase activity	THL incubation in COS cells	≥50% of luciferase activity of fresh THL	Pass
Purity	IgG	SDS-PAGE	Heavy and light chains with <5% impurity	Pass
	DNA	Agarose gel	Supercoil bands correspond to pGL4 standard	Pass
Safety	Endotoxin	LAL spectrophotometry	<5 EU/vial	2 EU/vial

*Following addition of a 40:1 ratio of HPγCD:phospholipid to the freshly prepared THLs, the solution was aliquoted to 2 mL/vial for lyophilization. HIR ED50 measured in the absence of Triton X-100. THL, Trojan horse liposome; PDI, polydispersity index; HIR, human insulin receptor; LAL, Limulus amoebocyte lysate; EU, endotoxin units. Reproduced with permission of Lee et al. (53)*.

#### Plasma Pharmacokinetics of Lyophilized THLs

THLs were lyophilized with HPγCD, followed by hydration and IV administration to Rhesus monkeys for a plasma pharmacokinetics evaluation. These THLs were produced with a recombinant form of the HIRMAb and encapsulated the pGL4.13 luciferase expression plasmid DNA (53). The THLs were lyophilized with a 40:1 molar ratio of HPγCD:phospholipid, followed by hydration, and IV administration in adult Rhesus monkeys at a low dose (12 ug/kg of THL encapsulated DNA) and a high dose (58 μg/kg of THL encapsulated DNA). Blood was removed for up to 3 h after the THL administration, and the rate of plasma clearance of the THL encapsulated DNA was determined using a mono-exponential pharmacokinetics analysis. The plasma concentration of the pGL4.13 plasmid DNA was determined by quantitative PCR using PCR primers specific for the luciferase open reading frame. The pGL4.13 plasmid served as the assay standard curve to enable conversion of Cq values into ng/mL of pGL4.13 DNA (53). The plasma clearance was constant at 0.9–1.1 mL/min/kg at either the high or low injection dose, and the rate of clearance of the hydrated freeze dried THLs was comparable to the plasma clearance of freshly prepared THLs (46).

#### Safety of Lyophilized THLs

Rhesus monkeys were observed for 48 h after the IV injection of hydrated freeze-dried THLs, and the animals showed no clinical signs of injection related reactions or other clinical signs. Over 25 tests of clinical chemistry, hematology, and coagulation were performed on blood removed pre-injection and 48 h post-injection, and there was no change in any parameter (53). These safety findings in the monkey corroborate previous studies, which demonstrated the safety of chronic administration of TfRMAb-targeted THLs in rats (297).

### Manufacturing and Market Demand

THL commercial manufacturing must be able to meet the market demand of a target diseases. Orphan diseases are most amenable to treatment with THLs owing to the small size of these markets. With respect to NPC1, it is estimated there are 2,000 patients with severe NPC1 in the seven major markets, 7MM (US, Japan, UK, France, Spain, Italy, and Germany). Assuming 50% penetration, then manufacturing must provide for drug for 1,000 patients per year treated weekly or 50 treatments per year. The target therapeutic dose of THL encapsulated DNA is 10 μg/kg, as this dose produces global expression of the LacZ gene in the Rhesus monkey brain (Figure 7) (50). Assuming an average body weight (BW) of the NPC1 child of 15 kg, then the weekly injection dose is 150 μg THL encapsulated DNA, which would be administered 50,000 times over the course of a year of weekly treatment of 1,000 patients. This would require manufacture of 7.5 g of encapsulated DNA. The ratio of MAb:plasmid DNA used in THL production is 2:1 (53). Therefore, 15 g of MAb would be needed per year to support manufacturing. The ratio of phospholipids:DNA in THL production is 20 μmol:1 mg (53). Therefore, 150 mmol of phospholipids would be required for annual production of THLs to meet market demand in the 7 MM for NPC1. Starting with equal 7.5 L volumes of 1 mg/mL of plasmid DNA and 7.5 L of ethanol/20 mM phospholipid (150 mmol), the amount of THLs required to meet market demand for an orphan disease such as NPC1 could be met in a single 15 L manufacturing run. The ethanol dilution method has previously been scaled up to the 5–10 L range for either small molecules (285) or plasmid DNA (281).

The treatment of orphan disease invariably involves *replacement gene therapy*, which requires lifelong weekly treatment. Meeting market demand for these rare diseases is feasible with reasonable starting volumes of materials, owing to the small number of orphan disease patients. For the non-orphan common brain diseases, it is not feasible to treat large numbers of patients with gene replacement therapy and weekly injections 52 weeks every year. However, it is possible to meet market demand for larger number of patients with short courses of *curative gene therapy*. For example, GBM might be cured with plasmid DNA that encodes multiple tandem expression cassettes for therapeutic genes, such as a gene encoding an shRNA against the EGFR (59), an shRNA or antisense RNA against the phosphate and tensin homolog, PTEN, and a gene knocking down the tumor suppressor p53 gene (298). Owing to the lack of size limitation of plasmid DNA that can be encapsulated in THLs (68), a single plasmid DNA could encode multiple therapeutic genes, each under the influence of specific promoters.

The prevalence of GBM is 30 cases per 1 million population (299). Therefore, there are about 20,000 cases in the US and EU. Assuming 50% market penetration, the goal is to treat 10,000 cases per year with a 1 month course of weekly treatment or four infusions per patient, which amounts to 40,000 weekly infusions per year. Given an injection dose (ID) of 10 μg/kg, and an average BW of 50 kg, the weekly ID is 500 μg of THL encapsulated plasmid DNA. This market demand could be met with 20 g of THL encapsulated plasmid DNA, 400 mmol of phospholipid, 40 g of MAb, and a starting volume of 20 L of DNA and 20 L of ethanol/phospholipid.

The prevalence of PD is about 900,000 patients in the US with the diagnosis of 60,000 new patients each year (300). PD would be amenable to THL gene therapy if curative gene therapy was developed such that only a single infusion per patient would be needed. The feasibility of providing a cure for new onset PD with GDNF gene therapy, using a TH promoter, has been demonstrated previously in experimental PD (246, 247). If THL gene therapy is initiated early in the course of early onset PD with a neurotrophin gene driven by a tissue specific gene promoter, then long-lasting therapeutic effects may be achieved with single THL administration (246, 247). Assuming the market of early onset PD was 100,000 cases in the US and EU, and each patient was administered a single dose of THL gene therapy, then the annual number of doses would be 100,000. Given a therapeutic ID of 10 ug/kg and a BW of 50 kg, the ID is 0.5 mg of THL encapsulated plasmid DNA. The annual requirement for THL encapsulated DNA is 50 g of DNA, 1000 mmol of ethanol/phospholipid, and 100 g of MAb in a starting volume of 50 L of plasmid DNA and 50 L of phospholipid.

The production of 7.5–50 g of plasmid DNA per year can be achieved with a single 3–20 L bioreactor, as the yield of antibiotic resistance-free (AF) plasmid DNA produced with sucrose selection is 2–3 g/L (301). Monoclonal antibody yields reach 8 kg of MAb per 2,000 L bioreactor (302). Therefore, the production of 15–100 g of MAb would require a single bioreactor volume of <12 L.

## Conclusions

Intravenous AAV9-based viral gene therapy is now FDA approved as a one-time treatment for juvenile spinal muscular atrophy (10). However, there are caveats to AAV gene therapy. First, the high dose of the AAV approved for human use, 2 × 10^14^ vg/kg, in SMA (10) may have genotoxic effects (11), and is associated with a high incidence of delayed hepatic cancer in mice (12). Conversely, the dose of THL encapsulated plasmid DNA in monkeys is 2 log orders of magnitude lower, 12 μg/kg, or 10^12^ vg/kg (Table 4). At this dose, no integration of plasmid DNA into the host chromosome is observed (256). Second, the strong immune response against the viral coat protein prevents a second injection of the virus, which limits AAV treatment to a single administration (303). THLs have low immunogenicity and are administered by chronic weekly IV administration (Table 4). Third, AAV9 delivery across the BBB from blood is inefficient and <30% of brain cells are transduced even at high injection doses, 4 × 10^13^ vg/kg, of scAAV9 (8, 9), whereas 100% of brain cells are transduced following IV administration of THLs (Table 4). Fourth, the size of the gene that can be inserted into the AAV genome is <2.3 kb for scAAV and <4.7 kb for ssAAV (9). In contrast, gene expression in brain *in vivo* is demonstrated following the IV administration of THLs encapsulating plasmid DNA >20 kb (Table 4). These limitations of viral gene therapy provide the rationale for the parallel development of non-viral targeted nanomedicines that deliver plasmid DNA across the BBB following IV administration. This review covers the multiple variables to be considered in the engineering of plasmid DNA nanomedicines that cross the BBB (Figure 1). First, the nanocontainer design, e.g., nanoparticle vs. liposome, must be chosen. Second, the type of BBB Trojan horse, e.g., peptide vs. MAb, as well as the specific receptor on the brain capillary endothelium that is targeted by the Trojan horse, must be considered. Third, the plasmid DNA must be engineered with tissue specific gene promoters, to eliminate off-target effects, and any plasmid DNA planned for human trials must be engineered without an antibiotic resistance selection gene. Fourth, both upstream and downstream manufacturing processes must be developed that meet the market demand for the target disease.

**Table 4:** Comparison of AAV and THL intravenous gene therapy of brain.

**Property**	**AAV**	**THL**	**References**
Immunogenicity	High	Low	(13, 297)
Frequency of administration	Single	Chronic	(54, 297, 303)
Transfection of brain cells	<30%	100%	(8, 9, 54, 256)
Injection dose (vg/kg)	10^14^	10^12^	(10, 50)
Size limitation of therapeutic gene	2–4 kb	>20 kb	(9, 68)
Chromosomal integration	At high doses	None	(11, 12, 256)

This review has focused on the development of THLs as receptor targeted nanomedicines for plasmid DNA delivery to brain. It is possible to encapsulate large size plasmid DNA, of at least 22 kb in size within THLs (68), which means the plasmid DNA can be engineered with large tissue specific promoters, such as the 8 kb TH promoter (246, 247). THLs have a proven safety record with chronic treatment in rats (297) and mice (54, 59), and hydrated/lyophilized THLs produce no injection related reactions and no changes in clinical chemistry in Rhesus monkeys (53). The IV administration of low dose, e.g., 12 μg/kg of THL encapsulated plasmid DNA, in Rhesus monkeys results in the delivery of 3–4 plasmid DNA molecules to all cells in the monkey brain (256), and global expression of the transgene throughout the brain of the primate (50). Off-target effects with THL gene therapy are eliminated with the use of tissue-specific gene promoters (Figure 7). THL manufacturing is scalable with the ethanol dilution method (Figure 10). Following lyoprotectant optimization, THLs can be lyophilized with HPγCD as the optimized lyoprotectant (Figure 11), stored for prolonged periods at 4°C, and hydrated with recovery of THL potency (53).

Targeted plasmid DNA nanomedicines for brain require the merger of multiple technologies, including liposomes, pegylation, receptor-specific Trojan horses, plasmid DNA engineering, scalable manufacturing, and lyophilization. Trojan horse nanomedicines for brain gene therapy, if approved for human use, will be one of the most technically advanced of the biologic human medicines.

## Author Contributions

The author confirms being the sole contributor of this work and has approved it for publication.

## Conflict of Interest

The author was the founder of The Lipogene Company, Inc.
